# Supplementary material for: Phosphorylation of Human Polyomavirus Large and Small T Antigens: An Ignored Research Field
Source: Viruses. 2023 Nov 9;15(11):2235. doi: 10.3390/v15112235 (PMC10674619; doi:10.3390/v15112235)
Supplement: Supplementary file 1 [file viruses-15-02235-s001.zip › viruses-2694256-supplementary.pdf]

Supplementary Table S1. Putative phosphorylation sites in SV40 and HPyV LTAg and their protein kinase. Putative protein kinases were predicted using the algorithm NetPhos3.1 [1,2]. Protein kinase consensus motifs are based on [3-7]. Proven phosphorylation sites are shown in grey.

residue	Protein kinase	Consensus motif	Sequence in LTAg	score
S10	PKA	K/RK/RXS/T	NREESLQLM	0.749
S22	CKI<br>CDK1	D/E(X)XS/T<br>LPXSPXKK	GLERSAWGN	0.510<br>0.503
T57	PKC	R/KR/KR/K/FSFRR	KKMNTLYKK	0.842
T85	CKII	D/ED/ED/ES/TD/ED/E	TEIPTYGTD	0.534
T88	CKII	D/ED/ED/ES/TD/ED/E	PTYGTDEWE	0.583
S111	CKII	D/ED/ED/ES/TD/ED/E	EEMPSSDDE	0.638
S112	CKII	D/ED/ED/ES/TD/ED/E	EMPSSDDEA	0.723
S120	ATM<br>CKI	L/SSQE/D<br>D/E(X)XS/T	ATADSQHST	0.556<br>0.549
S123	PKC	R/KR/KR/K/FSFRR	DSQHSTPPK	0.666
T124	PKC<br>CDK5<br>$p38^{MAPK}$<br>GSK3	R/KR/KR/K/FSFRR<br>S/TPXK/H/R<br>PXS/TP<br>S/TXXXS/T	SQHSTPPKK	0.727<br>0.603<br>0.523<br>0.502
T163	PKC	R/KR/KR/K/FSFRR	FAIYTTKEK	0.750
T182	PKC	R/KR/KR/K/FSFRR	KYSVTFISR	0.829
S189	PKA<br>RSK	K/RK/RXS/T<br>RXRXXS/T	SRHNSYNHN	0.606<br>0.515
T199	$p38^{MAPK}$<br>CDK5	PXS/TP<br>S/TPXK/H/R	LFFLTPHRH	0.567<br>0.535
S206	PKA<br>PKG	K/RK/RXS/T<br>R/KR/KXS/T	RHRVSAINN	0.748<br>0.556
T237	PKC	R/KR/KR/K/FSFRR	YSALTRDPF	0.762
S242	CKII<br>PKC	D/ED/ED/ES/TD/ED/E<br>R/KR/KR/K/FSFRR	RDPFSVIEE	0.617<br>0.586
S247	CKI<br>DNAPK	D/E(X)XS/T<br>QXS/TQE/D	VIEESLPGG	0.574<br>0.502
T265	CKI	D/E(X)XS/T	EAEETKQVS	0.516
S269	PKC	R/KR/KR/K/FSFRR	TKQVSWKV	0.597
T280	CKII	D/ED/ED/ES/TD/ED/E	YAMETKCDD	0.504
S330	PKC	R/KR/KR/K/FSFRR	IFADSKNQK	0.826
T343	PKC	R/KR/KR/K/FSFRR	QAVDTVLAK	0.844
S352	PKA	K/RK/RXS/T	KRVDSLQLT	0.835
T362	PKC	R/KR/KR/K/FSFRR	EQMLTNRFN	0.722
T379	CKII	D/ED/ED/ES/TD/ED/E	MFGSTGSAD	0.521
S381	CKII	D/ED/ED/ES/TD/ED/E	GSTGSADIE	0.566
Y414	EGFR	XXEXYYYXX	KCMVYNIPK	0.505
T434	PKC	R/KR/KR/K/FSFRR	SGKTTLAAA	0.593
T478	CKII	D/ED/ED/ES/TD/ED/E	DVKGTGGES	0.563
S482	CKII	D/ED/ED/ES/TD/ED/E	TGGESRDLP	0.503
S504	PKC	R/KR/KR/K/FSFRR	YLDGSVKVN	0.869
T518	PKG	R/KR/KXS/T	LNKRTQIFP	0.548
T527	CKII<br>PKG	D/ED/ED/ES/TD/ED/E<br>R/KR/KXS/T	PGIVTMNEY	0.520<br>0.517
Y531	SRC	EEDVYGXV	TMNEYSVPK	0.501
S560	CKII	D/ED/ED/ES/TD/ED/E	CLERSEFLL	0.528
S571	PKA<br>PKC	K/RK/RXS/T<br>R/KR/KR/K/FSFRR	RIIQSGIAL	0.804<br>0.698
S591	CDK1	LPXSPXKK	EFAQSIQSR	0.509
S594	PKC	R/KR/KR/K/FSFRR	QSIQSRIVE	0.601

S608	CKI	D/E(X)XS/T	DKEFSLSVY	0.518
S610	PKC	R/KR/KR/K/FSFRR	EFSLSVYQK	0.776
S632	CKII	D/ED/ED/ES/TD/ED/E	WLRNSDDDD	0.697
S639	CKII<br>CKI<br>DNAPK<br>ATM	D/ED/ED/ES/TD/ED/E<br>D/E(X)XS/T<br>QXS/TQE/D<br>L/SSQE/D	DDEDSQENA	0.648<br>0.606<br>0.603<br>0.599
S657	CKII	D/ED/ED/ES/TD/ED/E	NMEDSGHET	0.598
S661	CKII	D/ED/ED/ES/TD/ED/E	SGHETGIDS	0.567
S665	DNAPK<br>ATM	QXS/TQE/D<br>L/SSQE/D	TGIDSQSQG	0.635<br>0.577
S667	DNAPK<br>PKC<br>ATM<br>CDK1	QXS/TQE/D<br>R/KR/KR/K/FSFRR<br>L/SSQE/D<br>LPXSPXKK	IDSQSQGSF	0.604<br>0.601<br>0.585<br>0.520
S670	CDK1<br>PKC	LPXSPXKK<br>R/KR/KR/K/FSFRR	QSQGSFQAP	0.581<br>0.568
S677	DNAPK<br>CDK1<br>ATM	QXS/TQE/D<br>LPXSPXKK<br>L/SSQE/D	APQSSQSVH	0.637<br>0.560<br>0.534
S679	PKC	R/KR/KR/K/FSFRR	QSSQSVHDH	0.537
Y687	EGFR	XXEXYYYXX	HNQPYHICR	0.550
T694	PKC	R/KR/KR/K/FSFRR	CRGFTCFKK	0.757
T701	$p38^{MAPK}$<br>GSK3	PXS/TP<br>S/TXXXS/T	KKPPTPPPE	0.555<br>0.519

Prediction based on motifs for the protein kinases ATM, CDK1, CDK5, CKI, CKII, CaMKII, DNAPK, EGFR, GSK3, INSR, $p38^{MAPK}$, PKA, PKB, PKC, PKG, RSK, and SRC.

BKPyV

residue	Protein kinase	Consensus motif	Sequence in LTAg	score
T57	PKC	R/KR/KR/K/FSFRR	KRMVTLYKK	0.798
S78	CKII	D/ED/ED/ES/TD/ED/E	FGTWSSSEV	0.582
T84	CKII	D/ED/ED/ES/TD/ED/E	SEVPTYGTE	0.554
Y85	SRC	EEDVYGXV	EVGTYGTEE	0.531
T87	CKII	D/ED/ED/ES/TD/ED/E	PTYGTEEWE	0.547
S95	CKII	D/ED/ED/ES/TD/ED/E	ESWWSSFNE	0.511
S96	PKC<br>CKII<br>CKI<br>CDK1	R/KR/KR/K/FSFRR<br>D/ED/ED/ES/TD/ED/E<br>D/E(X)XS/T<br>LPXSPXKK	SWWSFNEK	0.563<br>0.541<br>0.529<br>0.507
S114	CKII	D/ED/ED/ES/TD/ED/E	DMFASDEEA	0.687
S122	ATM<br>CKI	L/SSQE/D<br>D/E(X)XS/T	ATADSQHST	0.555<br>0.550
S125	PKC	R/KR/KR/K/FSFRR	DSQHSTPPK	0.666
T126	PKC<br>CDK5<br>$p38^{MAPK}$<br>GSK3	R/KR/KR/K/FSFRR<br>S/TPXK/H/R<br>PXS/TP<br>S/TXXXS/T	SQHSTPPKK	0.727<br>0.603<br>0.523<br>0.502
S142	PKG	R/KR/KXS/T	KDFPSDLHQ	0.548
S149	PKC<br>DNAPK<br>ATM	R/KR/KR/K/FSFRR<br>QXS/TQE/D<br>L/SSQE/D	HQFLSQAVF	0.749<br>0.614<br>0.598
T165	PKC<br>PKG	R/KR/KR/K/FSFRR<br>R/KR/KXS/T	FAVYTTKEK	0.685<br>0.503

T166	PKC	R/KR/KR/K/FSFRR	AVYTTKEKA	0.541
T184	PKC	R/KR/KR/K/FSFRR	KYSVTFISR	0.829
T201	CDK5<br>$p38^{MAPK}$	S/TPXK/H/R<br>PXS/TP	IFFLTPHRH	0.579<br>0.572
S208	PKA<br>PKG	K/RK/RXS/T<br>R/KR/KXS/T	RHVSAINN	0.748<br>0.544
T239	PKC	R/KR/KR/K/FSFRR	YSALTRDPY	0.559
T245	CKII	D/ED/ED/ES/TD/ED/E	DPYHTIEES	0.590
S271	PKC	R/KR/KR/K/FSFRR	TKQVSWKLI	0.629
T282	CKII	D/ED/ED/ES/TD/ED/E	YAVETKCED	0.628
Y299	INSR	YXXM	LEFQYNVEE	0.505
S332	PKC	R/KR/KR/K/FSFRR	IFAESKNQK	0.845
S337	PKA	K/RK/RXS/T	KNQKSICQQ	0.506
T345	PKC	R/KR/KR/K/FSFRR	QAVDTVLAK	0.844
T354	PKA<br>PKG	K/RK/RXS/T<br>R/KR/KXS/T	KRVDTLHMT	0.669<br>0.551
T358	CKII	D/ED/ED/ES/TD/ED/E	TLHMTREEM	0.578
T364	PKC	R/KR/KR/K/FSFRR	EEMLTERFN	0.563
T435	PKC	R/KR/KR/K/FSFRR	DSGKTTLAA	0.543
T480	PKC<br>CKII	R/KR/KR/K/FSFRR<br>D/ED/ED/ES/TD/ED/E	DVKGTGAES	0.684<br>0.598
S484	CKII	D/ED/ED/ES/TD/ED/E	TGAESKDLP	0.546
S506	PKC<br>CDK1	R/KR/KR/K/FSFRR<br>LPXSPXKK	YLDGSVKVN	0.869<br>0.515
T520	PKG	R/KR/KXS/T	LNKRTQIFP	0.548
T529	PKG	R/KR/KXS/T	PGLVTMNEY	0.553
S558	PKA<br>RSK	K/RK/RXS/T<br>RXRXXS/T	YLRKSLNQS	0.605<br>0.501
S573	PKA	K/RK/RXS/T	RILQSGMTL	0.821
S596	CKII	D/ED/ED/ES/TD/ED/E	TDIQSRIVE	0.501
S607	PKA<br>RSK<br>PKG	K/RK/RXS/T<br>RXRXXS/T<br>R/KR/KXS/T	ERLDSEISM	0.695<br>0.551<br>0.509
S610	CDK1	LPXSPXKK	DSEISMYTF	0.515
T613	PKC	R/KR/KR/K/FSFRR	ISMYTFSRM	0.752
S615	PKC	R/KR/KR/K/FSFRR	MYTFSRMKY	0.658
T631	CKII	D/ED/ED/ES/TD/ED/E	ILDITREED	0.629
S636	CKII	D/ED/ED/ES/TD/ED/E	REEDSETED	0.687
T638	CKII<br>CKI<br>CDK1	D/ED/ED/ES/TD/ED/E<br>D/E(X)XS/T<br>LPXSPXKK	EDSETEDSG	0.591<br>0.532<br>0.526
S641	CKI	D/E(X)XS/T	ETEDSGHGS	0.569
S645	CKII	D/ED/ED/ES/TD/ED/E	SGHGSSTES	0.621
S646	CDK1	LPXSPXKK	GHGSSTESQ	0.515
T647	PKC<br>CDK1	R/KR/KR/K/FSFRR<br>LPXSPXKK	HGSSTESQS	0.639<br>0.551
S649	DNAPK<br>ATM	QXS/TQE/D<br>L/SSQE/D	SSTESQSQC	0.647<br>0.527
S651	DNAPK	QXS/TQE/D	TESQSQCSS	0.586
S654	CDK1	LPXSPXKK	QSQCSSQVS	0.552
S655	DNAPK	QXS/TQE/D	SQCSSQVSD	0.590
S667	DNAPK<br>ATM	QXS/TQE/D<br>L/SSQE/D	PAEDSQRSD	0.522<br>0.512
S674	DNAPK<br>ATM	QXS/TQE/D<br>L/SSQE/D	SDPHSQELH	0.634<br>0.623

T691	p38^{MAPK}<br>GSK3<br>CDK5	PXS/TP<br>S/TXXXS/T<br>S/TPXK/H/R	KRPKTPPPK	0.534<br>0.524<br>0.514

JCPyV

residue	Protein kinase	Consensus motif	Sequence in LTAg	score
T84	CKII	D/ED/ED/ES/TD/ED/E	SEVPTYGTD	0.509
T87	CKII	D/ED/ED/ES/TD/ED/E	PTVPTYGTD	0.566
T96	CKII	D/ED/ED/ES/TD/ED/E	SWWNTFNEK	0.534
S114	CKII	D/ED/ED/ES/TD/ED/E	EMFASDDEN	0.699
S121	DNAPK<br>ATM	QXS/TQE/D<br>L/SSQE/D	ENTGSQHST	0.634<br>0.627
S124	PKC	R/KR/KR/K/FSFRR	GSQHSTPPK	0.699
T125	CDK5<br>PKC<br>GSK3	S/TPXK/H/R<br>R/KR/KR/K/FSFRR<br>S/TXXXS/T	SQHSTPPKK	0.659<br>0.647<br>0.503
S148	PKC<br>DNAPK<br>ATM	R/KR/KR/K/FSFRR<br>QXS/TQE/D<br>L/SSQE/D	HAFLSQAVF	0.748<br>0.614<br>0.582
S159	PKC	R/KR/KR/K/FSFRR	RTVASFAVY	0.785
T164	PKC	R/KR/KR/K/FSFRR	FAVYTTKEK	0.685
T165	PKC	R/KR/KR/K/FSFRR	AVYTTKEKA	0.541
T183	PKC	R/KR/KR/K/FSFRR	KYSVTFISR	0.829
T200	p38^{MAPK}<br>CDK5	PXS/TP<br>S/TPXK/H/R	LEFLTPHRH	0.567<br>0.547
S207	PKA<br>PKG	K/RK/RXS/T<br>R/KR/KXS/T	RHRVSAINN	0.756<br>0.556
S248	CKI	D/E(X)XS/T	VVEESIQGG	0.544
S270	PKC<br>PKA	R/KR/KR/K/FSFRR<br>K/RK/RXS/T	TKQVSWKLV	0.597<br>0.505
T281	CKII	D/ED/ED/ES/TD/ED/E	YALETKCED	0.577
Y322	SRC	EEDVYGXV	HEKHYYNAQ	0.502
Y323	EGFR	XXEXYYYXX	EKHYYNAQI	0.554
S331	PKC	R/KR/KR/K/FSFRR	IFADSKNQK	0.722
T344	PKC	R/KR/KR/K/FSFRR	QAVDTVAAK	0.816
S353	PKA<br>PKG	K/RK/RXS/T<br>R/KR/KXS/T	QRVDSIHMT	0.598<br>0.514
T357	CKII	D/ED/ED/ES/TD/ED/E	SIHMTREEM	0.590
T435	PKC	R/KR/KR/K/FSFRR	SGKTTLAAA	0.593
T479	CKII	D/ED/ED/ES/TD/ED/E	DVKGTGAES	0.590
S483	CKII	D/ED/ED/ES/TD/ED/E	TGAESRDLP	0.536
S488	PKC	R/KR/KR/K/FSFRR	RDLPSGHGI	0.549
S493	CDK1	LPXSPXKK	GHGISNLDC	0.529
S505	PKC	R/KR/KR/K/FSFRR	YLDGSVKVN	0.569
T519	CDK1	LPXSPXKK	QNKRTQVFP	0.513
T528	CKII	D/ED/ED/ES/TD/ED/E	PGIVTMNEY	0.520
S533	CDK1	LPXSPXKK	MNEYSVPRT	0.516
S557	RSK	RXRXXS/T	YLRKSLSCS	0.541
S559	PKA	K/RK/RXS/T	RKSLSCSEY	0.575
S561	CKII	D/ED/ED/ES/TD/ED/E	SLSCSEYLL	0.521
S572	PKA	K/RK/RXS/T	RILQSGMTL	0.824
S609	CDK1	LPXSPXKK	DLEISMYTF	0.515
Y611	EGFR	XXEXYYYXX	EISMYTFST	0.515
S614	PKC	R/KR/KR/K/FSFRR	MYTFSTMKA	0.731

T615	PKC	R/KR/KR/K/FSFRR	YTFSTMKAN	0.911
S635	CKII	D/ED/ED/ES/TD/ED/E	REEDSEAED	0.699
S640	CKI	D/E(X)XS/T	EAEDSGHGS	0.542
S644	CKII	D/ED/ED/ES/TD/ED/E	SGHGSSTES	0.621
S645	CDK1	LPXSPXKK	GHGSSTESQ	0.505
T646	PKC	R/KR/KR/K/FSFRR	HGSSTESQS	0.639
S648	DNAPK	QXS/TQE/D	SSTESQSQC	0.647
S650	DNAPK	QXS/TQE/D	TESQSQCFS	0.586
S654	DNAPK	QXS/TQE/D	SQCFSQVSE	0.520
T664	ATM<br>CKII	L/SSQE/D<br>D/ED/ED/ES/TD/ED/E	SGADTQENC	0.517<br>0.513
T648	$p38^{MAPK}$<br>GSK3	PXS/TP<br>S/TXXXS/T	KKPKTPPPK	0.548<br>0.520

KIPyV

residue	Protein kinase	Consensus motif	Sequence in LTAg	score
S6	CKII	D/ED/ED/ES/TD/ED/E	DKTLTREEA	0.518
S65	CDK1	LPXSPXKK	KLQDSVSSV	0.514
S82	CDK1	LPXSPXKK	NIWQSSQIP	0.516
S83	DNAPK<br>ATM	QXS/TQE/D<br>L/SSQE/D	IWQSSQIPT	0.574<br>0533
T90	$p38^{MAPK}$	PXS/TP	PTYGTPDLD	0.540
S98	ATM<br>DNAPK	L/SSQE/D<br>QXS/TQE/D	DEWWSQFNT	0.633<br>0.605
T102	CKII	D/ED/ED/ES/TD/ED/E	SQFNTYWEE	0.699
S113	DNAPK	QXS/TQE/D	RCNESMPSS	0.543
S116	PKC	R/KR/KR/K/FSFRR	ESMPSSPKR	0.633
S117	CDK5<br>GSK3	S/TPXK/H/R<br>S/TXXXS/T	SMPSSPKRS	0.635<br>0.535
S121	PKG<br>CKII	R/KR/KXS/T<br>D/ED/ED/ES/TD/ED/E	SPKRSAPEE	0.612<br>0.590
S130	DNAPK<br>ATM	QXS/TQE/D<br>L/SSQE/D	EPSCSQATP	0.621<br>0.586
T133	$p38^{MAPK}$<br>CDK5	PXS/TP<br>S/TPXK/H/R	CSQATPPKK	0.565<br>0.525
S144	DNAPK	QXS/TQE/D	AFDASLEEP	0.558
S161	PKC	R/KR/KR/K/FSFRR	HAVFSNKCI	0.899
T172	PKC<br>CKII	R/KR/KR/K/FSFRR<br>D/ED/ED/ES/TD/ED/E	FVVHTTREK	0.836<br>0.521
S191	PKC	R/KR/KR/K/FSFRR	KYQCSFISK	0.778
T208	$p38^{MAPK}$<br>CDK5	PXS/TP<br>S/TPXK/H/R	IFFLTPHKH	0.585<br>0.539
S215	PKA<br>PKG	K/RK/RXS/T<br>R/KR/KXS/T	KHRVSAINN	0.758<br>0.569
S228	PKC	R/KR/KR/K/FSFRR	HCTVSFLFC	0.720
S303	PKC	R/KR/KR/K/FSFRR	YLLGSYIRF	0.771
T309	CKII	D/ED/ED/ES/TD/ED/E	IRFATKPEE	0.530
S318	CKII	D/ED/ED/ES/TD/ED/E	CEKCSKNDD	0.509
T324	PKC	R/KR/KR/K/FSFRR	NDDATHKRV	0.886
T350	DNAPK	QXS/TQE/D	KNACTQAID	0.525
Y362	EGFR	XXEXYYYXX	AERRYNCVT	0.522
T366	PKC	R/KR/KR/K/FSFRR	YNCVTLTRK	0.878
T368	PKC	R/KR/KR/K/FSFRR	CVTLTRKKL	0.883
T374	PKC	R/KR/KR/K/FSFRR	KKLLTKRFK	0.940

S400	PKA	K/RK/RXS/T	LYMASIAWY	0.510
T405	PKC	R/KR/KR/K/FSFRR	IAWYTGLNK	0.766
S440	PKA	K/RK/RXS/T	GPINSGKTT	0.528
S528	PKG	R/KR/KXS/T	VNKRSQIFP	0.514
T537	CKII<br>PKG	D/ED/ED/ES/TD/ED/E<br>R/KR/KXS/T	PGIVTMNEY	0.563<br>0.537
T546	PKC	R/KR/KR/K/FSFRR	CIPETVAVR	0.591
T558	PKC	R/KR/KR/K/FSFRR	TVMFTIKRN	0.883
S566	DNAPK	QXS/TQE/D	NLRESLEKT	0.532
T570	CDK5<br>GSK3	S/TPXK/H/R<br>S/TXXXS/T	SLEKTPQLL	0.649<br>0.501
S575	PKC<br>ATM	R/KR/KR/K/FSFRR<br>L/SSQE/D	PQLLSQRIL	0.650<br>0.518
S581	PKC<br>PKA	R/KR/KR/K/FSFRR<br>K/RK/RXS/T	RILHSGIAM	0.672<br>0.589
S596	CKII	D/ED/ED/ES/TD/ED/E	YRPVSDFDE	0.556
S604	CKII	D/ED/ED/ES/TD/ED/E	EEIQSNVVY	0.528
T620	PKC	R/KR/KR/K/FSFRR	YIGLTEFAT	0.590
T630	PKC	R/KR/KR/K/FSFRR	QMNVTNGKN	0.675

WU

residue	Protein kinase	Consensus motif	Sequence in LTAg	score
T4	CDK1	LPXSPXKK	MDKTLSRN	0.505
S6	PKC<br>CKII	R/KR/KR/K/FSFRR<br>D/ED/ED/ES/TD/ED/E	DKTLSRNEAA	0.621<br>0.547
T32	PKC	R/KR/KR/K/FSFRR	PLMRTKYLS	0.512
S36	PKC	R/KR/KR/K/FSFRR	TKYLSKCKE	0.638
S57	PKA<br>PKC	K/RK/RXS/T<br>R/KR/KR/K/FSFRR	KKLNSLYLK	0.681<br>0.620
S82	CDK1	LPXSPXKK	EVWSSSQIP	0.529
S83	DNAPK<br>ATM<br>CDK1	QXS/TQE/D<br>L/SSQE/D<br>LPXSPXKK	VWSSSQIPT	0.588<br>0.519<br>0.505
T90	CDK5<br>p38MAPK	S/TPXK/H/R<br>PXS/TP	PTYGTPDWD	0.552<br>0.506
Y95	EGFR	XXEXYYYXX	PDWDYWWSQ	0.550
S98	ATM<br>DNAPK	L/SSQE/D<br>QXS/TQE/D	DYWWSQFNS	0.639<br>0.605
S102	CKII	D/ED/ED/ES/TD/ED/E	SQFNSYWEE	0.701
S117	GSK3<br>CKI<br>p38MAPK	S/TXXXS/T<br>D/E(X)XS/T<br>PXS/TP	EMPKSPGET	0.540<br>0.522<br>0.519
T121	CDK5<br>p38MAPK	S/TPXK/H/R<br>PXS/TP	SPGETPTKR	0.591<br>0.522
T126	CKII	D/ED/ED/ES/TD/ED/E	PTKRTREDD	0.511
S136	DNAPK<br>ATM	QXS/TQE/D<br>L/SSQE/D	EPQCSQATP	0.612<br>0.537
T139	p38MAPK<br>CDK5	PXS/TP<br>S/TPXK/H/R	CSQATPPKK	0.571<br>0.515
S152	DNAPK	QXS/TQE/D	ATDASLSFP	0.561
S154	PKC	R/KR/KR/K/FSFRR	DASLSFPKE	0.670
S164	DNAPK	QXS/TQE/D	EEFVSQAVF	0.585

	ATM	L/SSQE/D		0.562
T180	PKC<br>CKII	R/KR/KR/K/FSFRR<br>D/ED/ED/ES/TD/ED/E	FVIHTTKEK	0.815<br>0.553
T181	PKC	R/KR/KR/K/FSFRR	VIHTTKEKA	0.525
T187	PKC	R/KR/KR/K/FSFRR	EKAETLYKK	0.759
S194	PKC	R/KR/KR/K/FSFRR	KKLLSKFKC	0.842
S202	PKG	R/KR/KXS/T	CNFASRHSY	0.543
S205	PKA<br>DNAPK	K/RK/RXS/T<br>QXS/TQE/D	ASRHSYYNT	0.830<br>0.552
T216	p38MAPK<br>CDK5	PXS/TP<br>S/TPXK/H/R	VFILTPFRH	0.654<br>0.507
S223	PKA	K/RK/RXS/T	RHRVSAVNN	0.725
Y247	EGFR	XXEXYYYXX	VNNAYGLYS	0.513
T254	PKC	R/KR/KR/K/FSFRR	YSRMTRDPF	0.542
T259	CKII	D/ED/ED/ES/TD/ED/E	RDPFTLCEE	0.620
Y280	SRC	EEDVYGXV	AEDLYGEFK	0.552
S293	CKII	D/ED/ED/ES/TD/ED/E	WKALSEFAL	0.518
S326	CDK1<br>CKII	LPXSPXKK<br>D/ED/ED/ES/TD/ED/E	EKCNSNEDA	0.539<br>0.507
S351	PKC	R/KR/KR/K/FSFRR	SDSKSQKNV	0.601
S371	PKA	K/RK/RXS/T	RRVDSLNMS	0.757
T394	PKC	R/KR/KR/K/FSFRR	KMDKTIKGE	0.681
T451	PKC	R/KR/KR/K/FSFRR	SGKTTVAAA	0.763
S509	CDK1	LPXSPXKK	GNGMSNLDN	0.547
S521	PKC	R/KR/KR/K/FSFRR	YLDGSVKVN	0.869
S535	PKG	R/KR/KXS/T	LNKRSQIFP	0.545
T544	PKG<br>CKII	R/KR/KXS/T<br>D/ED/ED/ES/TD/ED/E	PGIVTMNEY	0.546<br>0.506
T561	PKC	R/KR/KR/K/FSFRR	RFHKTVLFT	0.538
T565	PKC<br>p38MAPK	R/KR/KR/K/FSFRR<br>PXS/TP	TVLFTPKRH	0.783<br>0.533
S573	PKC	R/KR/KR/K/FSFRR	HLKESLDKT	0.807
T577	CDK5<br>GSK3<br>p38MAPK	S/TPXK/H/R<br>S/TXXXS/T<br>PXS/TP	SLDKTPELM	0.651<br>0.520<br>0.502
S588	PKC<br>PKA	R/KR/KR/K/FSFRR<br>K/RK/RXS/T	RVLQSGMCI	0.701<br>0.585
S603	PKA	K/RK/RXS/T	CRPVSDFHP	0.644
T627	PKC	R/KR/KR/K/FSFRR	YIGLTEFAD	0.534

MCPyV

residue	Protein kinase	Consensus motif	Sequence in LTAg	score
Y24	INSR	YXXM	APNCYGNIP	0.568
S37	PKC<br>PKG	R/KR/KR/K/FSFRR<br>R/KR/KXS/T	AFKRSCLKH	0.567<br>0.521
T57	PKC	R/KR/KR/K/FSFRR	MELNTLWSK	0.571
S60	PKC	R/KR/KR/K/FSFRR	NTLWSKFQQ	0.654
Y85	EGFR	XXEXYYYXX	EAPIYGTTK	0.524
T87	PKC	R/KR/KR/K/FSFRR	PIYGTTKFK	0.918
T88	PKC	R/KR/KR/K/FSFRR	IYGTTKFKE	0.660
S100	PKC<br>CKI	R/KR/KR/K/FSFRR<br>D/E(X)XS/T	SGGFSFGKA	0.687<br>0.542
S116	PKC	R/KR/KR/K/FSFRR	HGTNSRSRK	0.761
S118	CDK1	LPXSPXKK	TNSRSRKPS	0.508

S122	PKB<br>PKG<br>RSK	RXRX*X*S/TF/L<br>R/KR/KXS/T<br>RXRXXS/T	SRKPSSNAS	0.638<br>0.628<br>0.577
S123	PKC	R/KR/KR/K/FSFRR	RKPSSNASR	0.656
S126	CDK1	LPXSPXKK	SSNASRGAP	0.523
S131	CDK1	LPXSPXKK	RGAPSGSSP	0.521
S133	CDK1	LPXSPXKK	APSGSSPPH	0.601
S134	CDK5<br>CDK1<br>p38^{MAPK}<br>GSK3	S/TPXK/H/R<br>LPXSPXKK<br>PXS/TP<br>S/TXXXS/T	PSGSSPPHS	0.636<br>0.554<br>0.506<br>0.502
S138	DNAPK<br>GSK3	QXS/TQE/D<br>S/TXXXS/T	SPPHSQSSS	0.636<br>0.502
S140	CDK1	LPXSPXKK	PHSQSSSSG	0.591
S141	CKI	D/E(X)XS/T	HSQSSSSGY	0.563
S142	CDK1	LPXSPXKK	SQSSSSGYG	0.536
S147	PKC<br>CDK1	R/KR/KR/K/FSFRR<br>LPXSPXKK	SGYGSFSAS	0.741<br>0.507
S149	CDK1	LPXSPXKK	YGSFSASQA	0.596
S151	DNAPK<br>ATM<br>CDK1	QXS/TQE/D<br>L/SSQE/D<br>LPXSPXKK	SFSASQASD	0.637<br>0.622<br>0.531
S156	DNAPK<br>CDK1	QXS/TQE/D<br>LPXSPXKK	QASDSQSRG	0.598<br>0.559
S175	CDK1	LPXSPXKK	PTSSSGSSS	0.500
S177	CKII<br>CDK1<br>CKI	D/ED/ED/ES/TD/ED/E<br>LPXSPXKK<br>D/E(X)XS/T	SSSGSSSRE	0.536<br>0.516<br>0.513
S178	PKC<br>CKII	R/KR/KR/K/FSFRR<br>D/ED/ED/ES/TD/ED/E	SSGSSSREE	0.684<br>0.572
S179	CKII	D/ED/ED/ES/TD/ED/E	SGSSSREET	0.605
T184	CDK1	LPXSPXKK	REETTNSGR	0.531
S186	PKC	R/KR/KR/K/FSFRR	ETTNSGRES	0.581
S190	PKA	K/RK/RXS/T	SGRESSTPN	0.604
S191	PKA<br>RSK	K/RK/RXS/T<br>RXRXXS/T	GRESSTPNG	0.685<br>0.503
T192	CDK1	LPXSPXKK	RESSTPNGT	0.503
T196	CDK1	LPXSPXKK	TPNGTDVPR	0.503
S197	PKC	R/KR/KR/K/FSFRR	PNGTSVPRN	0.666
S202	PKA	K/RK/RXS/T	VPRNSSRTD	0.576
S203	PKA	K/RK/RXS/T	PRNSSRTDG	0.671
T205	CKII	D/ED/ED/ES/TD/ED/E	NSSRTDGTW	0.508
S217	DNAPK<br>CDK1	QXS/TQE/D<br>LPXSPXKK	FCDESLSSP	0.570<br>0.528
S219	CKII<br>CDK1	D/ED/ED/ES/TD/ED/E<br>LPXSPXKK	DESLSSPEP	0.628<br>0.524
S220	p38^{MAPK}	PXS/TP	ESLSSPEPP	0.535
S225	CKII	D/ED/ED/ES/TD/ED/E	PEPPSSSEE	0.673
S226	CKII	D/ED/ED/ES/TD/ED/E	EPPSSSEEP	0.664
S227	CKII	D/ED/ED/ES/TD/ED/E	PPSSSEEPE	0.620
S235	GSK3	S/TXXXS/T	EEPPSSRSS	0.507
S238	CDK1	LPXSPXKK	PSSRSSPRQ	0.553
S239	CDK5<br>p38^{MAPK}<br>GSK3<br>PKC	S/TPXK/H/R<br>PXS/TP<br>S/TXXXS/T<br>R/KR/KR/K/FSFRR	SSRSSPRQP	0.717<br>0.579<br>0.516<br>0.505

S245	CDK1	LPXSPXKK	RQPPSSSAE	0.577
S246	CKII	D/ED/ED/ES/TD/ED/E	QPPSSSAEE	0.618
S247	CDK1	LPXSPXKK	PPSSSAEEA	0.543
S253	CKII<br>CDK1	D/ED/ED/ES/TD/ED/E<br>LPXSPXKK	EEASSSQFT	0.520<br>0.520
S254	ATM<br>CKII<br>DNAPK<br>CKI<br>CDK1	L/SSQE/D<br>D/ED/ED/ES/TD/ED/E<br>QXS/TQE/D<br>D/E(X)XS/T<br>LPXSPXKK	EASSSQFTD	0.605<br>0.575<br>0.564<br>0.552<br>0.505
T257	CKII	D/ED/ED/ES/TD/ED/E	SSQFTDEEY	0.600
Y261	SRC	EEDVYGXV	TDEEYRSSS	0.541
S263	CKII	D/ED/ED/ES/TD/ED/E	EEYRSSSFT	0.502
S264	CKI<br>PKA	D/E(X)XS/T<br>K/RK/RXS/T	EYRSSSFTT	0.524<br>0.510
S265	CKI<br>PKA	D/E(X)XS/T<br>K/RK/RXS/T	YRSSSFTTP	0.590<br>0.578
T267	PKC	R/KR/KR/K/FSFRR	SSSFTTPKT	0.886
T268	CDK5<br>$p38^{MAPK}$	S/TPXK/H/R<br>PXS/TP	SSFTTPKTP	0.678<br>0.514
T271	CDK5<br>$p38^{MAPK}$<br>GSK3	S/TPXK/H/R<br>PXS/TP<br>S/TXXXS/T	TTPKTPPPF	0.610<br>0.526<br>0.510
S276	PKC	R/KR/KR/K/FSFRR	PPPFSRKRK	0.829
S284	PKC<br>CDK1	R/KR/KR/K/FSFRR<br>LPXSPXKK	KFGGSRSSA	0.632<br>0.527
S287	CDK1	LPXSPXKK	GSRSSASSA	0.554
S290	PKC	R/KR/KR/K/FSFRR	SSASSASSA	0.623
S292	CDK1	LPXSPXKK	ASSASSASF	0.527
S293	CDK1	LPXSPXKK	SSASSASFT	0.527
T297	PKC	R/KR/KR/K/FSFRR	SASFTSTTP	0.681
T299	CDK5<br>PKC<br>$p38^{MAPK}$	S/TPXK/H/R<br>R/KR/KR/K/FSFRR<br>PXS/TP	SFTSTPPKP	0.748<br>0.729<br>0.506
S320	CKII	D/ED/ED/ES/TD/ED/E	PIDLSDYLS	0.509
S324	PKC	R/KR/KR/K/FSFRR	SDYLSHAVY	0.619
S329	PKC	R/KR/KR/K/FSFRR	HAVYSNKTV	0.721
T341	CKII	D/ED/ED/ES/TD/ED/E	AIYTTSDKA	0.543
S342	PKC<br>CKII	R/KR/KR/K/FSFRR<br>D/ED/ED/ES/TD/ED/E	IYTTSDKAI	0.852<br>0.504
T375	CDK1	LPXSPXKK	LLFITLSKH	0.514
S377	PKC<br>CDK1	R/KR/KR/K/FSFRR<br>LPXSPXKK	FITLSKHRV	0.842<br>0.502
S382	PKA<br>PKG<br>PKC	K/RK/RXS/T<br>R/KR/KXS/T<br>R/KR/KR/K/FSFRR	KHRVSAIKN	0.693<br>0.566<br>0.514
T390	PKC	R/KR/KR/K/FSFRR	NFCSTFCTI	0.567
S501	PKC	R/KR/KR/K/FSFRR	LFYESKSQK	0.712
T514	PKC<br>CDK1	R/KR/KR/K/FSFRR<br>LPXSPXKK	QAADTVLAK	0.697<br>0.501
T527	CKII	D/ED/ED/ES/TD/ED/E	MLEMTRTEM	0.513
T580	PKC	R/KR/KR/K/FSFRR	IQLLTENIP	0.619
S601	PKC	R/KR/KR/K/FSFRR	SGKTSFAAA	0.614
S647	DNAPK	QXS/TQE/D	KGQNSLNKD	0.514
S675	CDK1	LPXSPXKK	AVAVSLEKK	0.503
T694	PKG	R/KR/KXS/T	PCIVTANDY	0.627

T703	PKC	R/KR/KR/K/FSFRR	FIPKTLIAR	0.652
S709	PKC	R/KR/KR/K/FSFRR	IARFSYTLH	0.582
T711	PKC	R/KR/KR/K/FSFRR	RFSYTLHFS	0.698
S723	PKA	K/RK/RXS/T	NLRDSLDQN	0.576
S738	PKA	K/RK/RXS/T	RILQSGTTL	0.682
T741	CDK1	LPXSPXKK	QSGTTLLLC	0.549
S772	PKC	R/KR/KR/K/FSFRR	QILQSEISY	0.788
T806	CKII<br>CKI	D/ED/ED/ES/TD/ED/E<br>D/E(X)XS/T	GPEETEETQ	0.608<br>0.606
T809	DNAPK<br>CKI	QXS/TQE/D<br>D/E(X)XS/T	ETEETQDSG	0.597<br>0.520
S812	CKI	D/E(X)XS/T	ETQDSGTFS	0.506
T814	PKC	R/KR/KR/K/FSFRR	QDSGTFSQ	0.705
S816	ATM<br>DNAPK	L/SSQE/D<br>QXS/TQE/D	SGTFSQ	0.663<br>0.620

*=polair amino acids

HPyV6

residue	Protein kinase	Consensus motif	Sequence in LTAg	score
S20	PKA<br>CDK1	K/RK/RXS/T<br>LPXSPXKK	LIGLSMACW	0.631<br>0.532
T65	PKC	R/KR/KR/K/FSFRR	KLNATLRDQ	0.572
S73	ATM	L/SSQE/D	QMSSSPTWC	0.520
T75	PKC<br>CDK1	R/KR/KR/K/FSFRR<br>LPXSPXKK	SSSPTWCFS	0.661<br>0.503
S90	$p38^{MAPK}$<br>GSK3<br>CDK5	PXS/TP<br>S/TXXXS/T<br>S/TPXK/H/R	PQYGSPGWE	0.538<br>0.508<br>0.502
Y110	SRC	EEDVYGXV	DEDLYCDEH	0.546
S116	CKII	D/ED/ED/ES/TD/ED/E	DEHLSASEE	0.731
S118	CKII	D/ED/ED/ES/TD/ED/E	HLSASEEED	0.704
S131	ATM<br>DNAPK	L/SSQE/D<br>QXS/TQE/D	GEGNSQDSK	0.623<br>0.616
T139	CDK5<br>$p38^{MAPK}$	S/TPXK/H/R<br>PXS/TP	KYSCTPPKK	0.583<br>0.552
T178	CKII<br>CKI	D/ED/ED/ES/TD/ED/E<br>D/E(X)XS/T	FVSYTTLEK	0.594<br>0.538
T179	CKI	D/E(X)XS/T	VSYTTLEKW	0.510
T185	DNAPK	QXS/TQE/D	EKWETLYDK	0.560
Y187	SRC	EEDVYGXV	WETLYDKLQ	0.505
T199	PKC	R/KR/KR/K/FSFRR	NAVFTGAYK	0.721
T216	$p38^{MAPK}$<br>CDK5	PXS/TP<br>S/TPXK/H/R	LYCITPRRH	0.634<br>0.531
S223	PKA	K/RK/RXS/T	RHRVSAMLN	0.721
S246	PKG	R/KR/KXS/T	AVLKSAECY	0.506
S264	CKII	D/ED/ED/ES/TD/ED/E	VIQESKAEG	0.581
S271	CKII<br>CDK1	D/ED/ED/ES/TD/ED/E<br>LPXSPXKK	EGLHSYDFQ	0.546<br>0.503
S278	CKII<br>PKC	D/ED/ED/ES/TD/ED/E<br>R/KR/KR/K/FSFRR	FQEGSKKEE	0.668<br>0.554
S290	PKC	R/KR/KR/K/FSFRR	NQVASFASD	0.667
T295	CKII	D/ED/ED/ES/TD/ED/E	FASDTDLTD	0.548
S316	PKC	R/KR/KR/K/FSFRR	NDPASCMKC	0.714

S368	PKC	R/KR/KR/K/FSFRR	LMLESTRQD	0.595
T396	CDK1	LPXSPXKK	GVEITQLLG	0.520
T413	PKC	R/KR/KR/K/FSFRR	QPSFTTKLK	0.791
T414	CDK1	LPXSPXKK	PSFTTKLKE	0.538
S421	CKII	D/ED/ED/ES/TD/ED/E	KEILSILTE	0.503
T424	CKI	D/E(X)XS/T	LSILTENIP	0.506
T445	PKC	R/KR/KR/K/FSFRR	SGKTTLAAA	0.543
T464	$p38^{MAPK}$	PXS/TP	NVNCTPDKI	0.510
T489	$p38^{MAPK}$	PXS/TP	DVKGTPMAN	0.536
T497	DNAPK	QXS/TQE/D	NTNLTQGCG	0.579
T503	CDK1	LPXSPXKK	GCGMTNLDN	0.517
T538	PKG	R/KR/KXS/T	PSVITCNEY	0.508
T547	PKC	R/KR/KR/K/FSFRR	VIPCTVKAR	0.898
T575	PKC	R/KR/KR/K/FSFRR	CVLMSKRLL	0.716
T583	CDK1	LPXSPXKK	LQKGTTLLA	0.529
S618	PKA	K/RK/RXS/T	ERWVSFGMY	0.716
T624	PKC<br>CKII	R/KR/KR/K/FSFRR<br>D/ED/ED/ES/TD/ED/E	GMYQTMKEN	0.781<br>0.536
T637	CKII	D/ED/ED/ES/TD/ED/E	IDPFTNVLV	0.513
T654	CKII	D/ED/ED/ES/TD/ED/E	ENDETNDST	0.505
S657	CKII	D/ED/ED/ES/TD/ED/E	ETNDSTQES	0.583
T658	DNAPK<br>ATM<br>CDK1	QXS/TQE/D<br>L/SSQE/D<br>LPXSPXKK	TNDSTQESG	0.636<br>0.577<br>0.512
S661	CKI	D/E(X)XS/T	STQESGIGS	0.510
S665	DNAPK	QXS/TQE/D	SGIGSMHSM	0.518

HPyV7

residue	Protein kinase	Consensus motif	Sequence in LTAg	score
S38	PKC<br>PKA	R/KR/KR/K/FSFRR<br>K/RK/RXS/T	VRLASKKYH	0.921<br>0.508
S73	RSK<br>$p38^{MAPK}$	RXRXXS/T<br>PXS/TP	QRSGSPMWH	0.537<br>0.500
S79	CKII	D/ED/ED/ES/TD/ED/E	MWHYSSDEV	0.572
S80	CKII	D/ED/ED/ES/TD/ED/E	WHYSSDEVR	0.504
S91	CDK5<br>$p38^{MAPK}$<br>GSK3	S/TPXK/H/R<br>PXS/TP<br>S/TXXXS/T	PPYGSPAWD	0.587<br>0.524<br>0.508
Y110	SRC	EEDVYGXV	DEDLYCTEE	0.539
T112	CKII	D/ED/ED/ES/TD/ED/E	DLYCTEELS	0.564
S116	CKII	D/ED/ED/ES/TD/ED/E	TEELSSSDE	0.708
S117	CKII	D/ED/ED/ES/TD/ED/E	EELSSSDEE	0.753
S118	CKII<br>CDK1	D/ED/ED/ES/TD/ED/E<br>LPXSPXKK	ELSSSDEEE	0.733<br>0.510
S136	ATM<br>DNAPK	L/SSQE/D<br>QXS/TQE/D	EEGCSQDSK	0.598<br>0.531
S142	PKC	R/KR/KR/K/FSFRR	DSKYSATPP	0.515
T144	$p38^{MAPK}$<br>CDK5	PXS/TP<br>S/TPXK/H/R	KYSATPPKQ	0.549<br>0.543
S163	CKII	D/ED/ED/ES/TD/ED/E	PECLSEFLS	0-505
T183	CKII	D/ED/ED/ES/TD/ED/E	FLCYTTYEK	0.505
Y207	EGFR	XXEXYYYXX	FIGAYNCVD	0.523
S213	PKA	K/RK/RXS/T	CVDSGALV	0.568
S221	CDK1	LPXSPXKK	VFFISGSRH	0.523

S223	PKC<br>CDK1	R/KR/KR/K/FSFRR<br>LPXSPXKK	FISGSRHRV	0.698<br>0.546
S228	PKA	K/RK/RXS/T	RHRVSAILN	0.732
T239	PKC	R/KR/KR/K/FSFRR	KKHCTVSFI	0.505
S241	PKC	R/KR/KR/K/FSFRR	HCTVSFIMV	0.850
S276	CKII<br>CDK1	D/ED/ED/ES/TD/ED/E<br>LPXSPXKK	GGLHSYDFQ	0.560<br>0.533
S283	PKC	R/KR/KR/K/FSFRR	FQEASKKDD	0.518
T303	CKII	D/ED/ED/ES/TD/ED/E	DMELTDVLL	0.507
S328	PKC	R/KR/KR/K/FSFRR	KCLKSVKAH	0.913
T347	PKC	R/KR/KR/K/FSFRR	KLKFTAKNQ	0.780
S373	PKC	R/KR/KR/K/FSFRR	LMMESTRKD	0.571
S382	PKC	R/KR/KR/K/FSFRR	LMVMSFKKQ	0.753
T401	CDK1	LPXSPXKK	GVEITQLIG	0.524
S426	CKII	D/ED/ED/ES/TD/ED/E	KEMLSYLVE	0.525
Y427	INSR	YXXM	EMLSYLVEN	0.500
T450	PKC	R/KR/KR/K/FSFRR	SGKTTLAAA	0.543
S470	PKC	R/KR/KR/K/FSFRR	VNCSSDKIN	0.611
T494	p38MAPK	PXS/TP	DVKGTPLPN	0.524
T533	PKC	R/KR/KR/K/FSFRR	HINKTSQLF	0.734
S534	CDK1	LPXSPXKK	INKTSQLFP	0.542
T543	PKG<br>CKII	R/KR/KXS/T<br>D/ED/ED/ES/TD/ED/E	PCIITCNEY	0.609<br>0.521
Y547	INSR	YXXM	TCNEYAIPT	0.502
T551	PKC	R/KR/KR/K/FSFRR	YAIPTTVKA	0.621
T552	PKC	R/KR/KR/K/FSFRR	AIPTTVKAR	0.861
S602	CKII	D/ED/ED/ES/TD/ED/E	WEPVSDFVE	0.645
S624	PKC	R/KR/KR/K/FSFRR	EQWVSYGMF	0.543
S630	PKC	R/KR/KR/K/FSFRR	GMFQTMKEN	0.692
S637	PKC	R/KR/KR/K/FSFRR	ENILSGKDP	0.506
S655	CKII	D/ED/ED/ES/TD/ED/E	TEENTRETQ	0.507
T658	DNAPK	QXS/TQE/D	NTRETQEST	0.617
S661	CKI	D/E(X)XS/T	ETQESTESG	0.593
T662	CKI	D/E(X)XS/T	TQESTESGI	0.530
S664	CKI<br>CDK1	D/E(X)XS/T<br>LPXSPXKK	ESTESGIGS	0.600<br>0.508
S668	DNAPK	QXS/TQE/D	SGIGSMNN	0.599

TSPyV

residue	Protein kinase	Consensus motif	Sequence in LTAg	score
S6	CKII<br>CKI	D/ED/ED/ES/TD/ED/E<br>D/E(X)XS/T	DKFLSREES	0.598<br>0.577
S10	PKA<br>DNAPK	K/RK/RXS/T<br>QXS/TQE/D	SREESLELM	0.660<br>0.503
S38	PKC<br>PKA	R/KR/KR/K/FSFRR<br>K/RK/RXS/T	HKKMSLKYH	0.921<br>0.565
S53	PKA	K/RK/RXS/T	PEKMSRLNQ	0.611
Y67	EGFR<br>SRC	XXEXYYYXX<br>EEDVYGXV	QEGIYNARQ	0.629<br>0.513
S76	CDK1	LPXSPXKK	EFPTSFSSQ	0.519
S79	ATM<br>DNAPK	L/SSQE/D<br>QXS/TQE/D	TSFSSQHDV	0.602<br>0.571

	CKI	D/E(X)XS/T		0.549
T85	DNAPK	QXS/TQE/D	HDVPTQDGR	0.552
S98	PKA	K/RK/RXS/T	YGHPSWASW	0.502
S127	CKII	D/ED/ED/ES/TD/ED/E	FCHESTIPS	0.514
S136	CDK5<br>$p38^{MAPK}$<br>GSK3	S/TPXK/H/R<br>PXS/TP<br>S/TXXXS/T	DESRSPSPT	0.539<br>0.521<br>0.502
S138	CDK5<br>GSK3<br>$p38^{MAPK}$	S/TPXK/H/R<br>S/TXXXS/T<br>PXS/TP	SRSPSPTPG	0.725<br>0.520<br>0.503
T140	CDK5<br>GSK3	S/TPXK/H/R<br>S/TXXXS/T	SPSPTPGPS	0.651<br>0.540
T145	ATM	L/SSQE/D	PGPSTQFSE	0.553
S163	GSK3	S/TXXXS/T	PPEDSPGCT	0.503
S170	PKC	R/KR/KR/K/FSFRR	CTQSSFSAT	0.552
T174	CDK5<br>$p38^{MAPK}$	S/TPXK/H/R<br>PXS/TP	SFSATPPKP	0.699<br>0.567
S181	PKG<br>PKA	R/KR/KXS/T<br>K/RK/RXS/T	KPKKSKYDS	0.637<br>0.525
S209	PKA	K/RK/RXS/T	NKTLSSFLI	0.542
S210	PKC<br>PKA	R/KR/KR/K/FSFRR<br>K/RK/RXS/T	KTLSSFLIY	0.752<br>0.502
T215	CKII	D/ED/ED/ES/TD/ED/E	FLIYTNEK	0.584
T216	CDK1<br>CKII	LPXSPXKK<br>D/ED/ED/ES/TD/ED/E	LIYTTNEKA	0.538<br>0.533
S239	PKA	K/RK/RXS/T	KSRHSFQEG	0.625
T250	$p38^{MAPK}$<br>CDK5	PXS/TP<br>S/TPXK/H/R	VFLMTPGKH	0.628<br>0.589
S257	PKA	K/RK/RXS/T	KHRVSAIKN	0.715
S270	PKC	R/KR/KR/K/FSFRR	HCTVSFLIC	0.745
S298	CKI	D/E(X)XS/T	LLEESKPGI	0.566
T322	PKC	R/KR/KR/K/FSFRR	WNLLTDFAV	0.595
S351	PKC	R/KR/KR/K/FSFRR	PSICSKCTK	0.654
T354	PKC	R/KR/KR/K/FSFRR	CSKCTKKAL	0.908
T380	PKC	R/KR/KR/K/FSFRR	KECKTQKTA	0.524
T383	PKG	R/KR/KXS/T	KTQKTACQQ	0.539
S403	PKC	R/KR/KR/K/FSFRR	KLIESTRKE	0.759
T421	CKII	D/ED/ED/ES/TD/ED/E	FEKLTDEFG	0.527
S440	CKII	D/ED/ED/ES/TD/ED/E	VAWYSCLFE	0.515
T479	PKC	R/KR/KR/K/FSFRR	SGKTTFAAA	0.704
S537	CDK1	LPXSPXKK	GQGVSNLDN	0.532
S549	PKC	R/KR/KR/K/FSFRR	HLDGSVKVD	0.870
S563	PKG	R/KR/KXS/T	VNKRSQIFP	0.514
T572	PKG	R/KR/KXS/T	PCLVTMNEY	0.565
T593	$p38^{MAPK}$<br>CDK1	PXS/TP<br>LPXSPXKK	VLNFTPKHN	0.577<br>0.511
S600	PKC	R/KR/KR/K/FSFRR	HNLRSCLQV	0.622
S605	PKA<br>CKII	K/RK/RXS/T<br>D/ED/ED/ES/TD/ED/E	CLQVSDYLL	0.649<br>0.510
Y607	EGFR	XXEXYYYXX	QVSDYLLTE	0.583
T610	PKC	R/KR/KR/K/FSFRR	DYLLTERIL	0.534
S636	CKII	D/ED/ED/ES/TD/ED/E	MFSESIKED	0.575
Y643	INSR	YXXM	EDVKYWKDI	0.511
S687	CKII<br>CKI	D/ED/ED/ES/TD/ED/E<br>D/E(X)XS/T	EEEFSETND	0.610<br>0.603

S689	CKII	D/ED/ED/ES/TD/ED/E	EFSETNDSG	0.530
S696	ATM	L/SSQE/D	SGFQTQ	0.523

HPyV9

residue	Protein kinase	Consensus motif	Sequence in LTAg	score
S6	CKII<br>DNAPK	D/ED/ED/ES/TD/ED/E<br>QXS/TQE/D	DQTLSLEEK	0.622<br>0.505
S28	PKC	R/KR/KR/K/FSFRR	WGNLSLMKK	0.521
Y34	EGFR	XXEXYYYXX	MKKAYKTVS	0.515
T36	PKC	R/KR/KR/K/FSFRR	KAYKTVSKI	0.831
S38	PKC	R/KR/KR/K/FSFRR	YKTVSKIYH	0.837
S75	PKC	R/KR/KR/K/FSFRR	SNCGSSSSQ	0.590
S76	CDK1	LPXSPXKK	NCGSSSQG	0.607
S77	CDK1	LPXSPXKK	CGSSSSQGY	0.503
S78	ATM<br>DNAPK<br>CDK1<br>CKI	L/SSQE/D<br>QXS/TQE/D<br>LPXSPXKK<br>D/E(X)XS/T	GSSSSQGYY	0.640<br>0.627<br>0.546<br>0.504
S83	CDK1<br>CKII<br>PKG	LPXSPXKK<br>D/ED/ED/ES/TD/ED/E<br>R/KR/KXS/T	QGYYSDSPY	0.544<br>0.508<br>0.504
S85	CDK1	LPXSPXKK	YYSDSPYFT	0.558
T91	CDK5<br>$p38^{MAPK}$	S/TPXK/H/R<br>PXS/TP	YFTETPFSY	0.613<br>0.561
S94	CKII	D/ED/ED/ES/TD/ED/E	ETPFSYCER	0.547
S130	CKII	D/ED/ED/ES/TD/ED/E	SETISSSDD	0.627
S131	CKII	D/ED/ED/ES/TD/ED/E	ETISSSDDE	0.620
S132	CKII	D/ED/ED/ES/TD/ED/E	TISSSDDEN	0.710
S149	CKII	D/ED/ED/ES/TD/ED/E	PSSASASED	0.510
S151	CKII	D/ED/ED/ES/TD/ED/E	SASASEDPD	0.624
S163	DNAPK<br>CDK1<br>ATM	QXS/TQE/D<br>LPXSPXKK<br>L/SSQE/D	EAGSSQSSF	0.614<br>0.559<br>0.520
S166	CKI	D/E(X)XS/T	SSQSSFTCT	0.545
T168	PKC	R/KR/KR/K/FSFRR	QSSFTCTPP	0.585
T170	CDK5<br>PKC<br>$p38^{MAPK}$	S/TPXK/H/R<br>R/KR/KR/K/FSFRR<br>PXS/TP	SFTCTPPKR	0.759<br>0.626<br>0.551
T181	CDK5<br>GSK3	S/TPXK/H/R<br>S/TXXXS/T	PEPNTPEDF	0.548<br>0.516
S199	PKC	R/KR/KR/K/FSFRR	HAIYSNKTM	0.688
T210	CKII	D/ED/ED/ES/TD/ED/E	FLIYTTVEK	0.514
T211	PKC	R/KR/KR/K/FSFRR	LIYTTVEKS	0.792
T221	PKC	R/KR/KR/K/FSFRR	QLYRTVEKS	0.822
S258	PKA<br>PKG	K/RK/RXS/T<br>R/KR/KXS/T	KHRVSAVKH	0.655<br>0.532
S271	PKC	R/KR/KR/K/FSFRR	QCTFSFIHC	0.794
T331	PKA	K/RK/RXS/T	EAKITDVLL	0.531
S351	PKC	R/KR/KR/K/FSFRR	PGTCSKCEK	0.667
S357	PKC<br>PKG	R/KR/KR/K/FSFRR<br>R/KR/KXS/T	CEKKSHKFH	0.664<br>0.560
S378	PKC	R/KR/KR/K/FSFRR	LFLESKSQK	0.619
S380	ATM	L/SSQE/D	LESKSQKNI	0.512
T404	CKII	D/ED/ED/ES/TD/ED/E	LVECTRMEL	0.543

	PKA	K/RK/RXS/T		0.516
S435	PKA<br>CDK1	K/RK/RXS/T<br>LPXSPXKK	LRWMSGVAW	0.818<br>0.512
T459	PKC	R/KR/KR/K/FSFRR	LQLVTTSQP	0.752
T460	PKC	R/KR/KR/K/FSFRR	QLVTTSQPK	0.533
S461	DNAPK	QXS/TQE/D	LVTTSQPKK	0.631
S476	PKA	K/RK/RXS/T	GPINSGKTT	0.532
T480	PKC	R/KR/KR/K/FSFRR	SGKTTLASA	0.512
S550	PKC	R/KR/KR/K/FSFRR	HLDGTIKVN	0.873
S564	PKG	R/KR/KXS/T	VNKRSQIFP	0.514
T573	PKG<br>DNAPK	R/KR/KXS/T<br>QXS/TQE/D	PVIMTMNEY	0.593<br>0.509
T597	PKC	R/KR/KR/K/FSFRR	FHCKTYLKQ	0.700
S606	CKII<br>CDK1	D/ED/ED/ES/TD/ED/E<br>LPXSPXKK	SLEKSDLIE	0.552<br>0.531
S616	PKA	K/RK/RXS/T	RILNSGYTI	0.768
Y618	EGFR	XXEXYYYXX	LNSGYTILL	0.512
T634	$p38^{MAPK}$	PXS/TP	VDSFTPKVQ	0.519
S653	PKA	K/RK/RXS/T	ERHVSITQF	0.730
T655	ATM	L/SSQE/D	HVSITQFGN	0.534

HPyV10

residue	Protein kinase	Consensus motif	Sequence in LTAg	score
S6	CKII<br>RSK<br>CKI	D/ED/ED/ES/TD/ED/E<br>RXRXXS/T<br>D/E(X)XS/T	DRVLSRDEV	0.576<br>0.540<br>0.502
T37	PKC	R/KR/KR/K/FSFRR	KYRQTCLKL	0.696
S60	PKC	R/KR/KR/K/FSFRR	NELFSKMYT	0.531
Y63	INSR	YXXM	FSKMYTTIE	0.522
T64	PKC	R/KR/KR/K/FSFRR	SKMYTTIEK	0.639
Y76	SRC	EEDVYGXV	EGEVYFPAK	0.516
Y85	EGFR<br>SRC	XXEXYYYXX<br>EEDVYGXV	GNPTYGTPE	0.573<br>0.523
T87	$p38^{MAPK}$	PXS/TP	PTYGTPEWD	0.530
S106	CKII	D/ED/ED/ES/TD/ED/E	DEDLSCNES	0.539
S114	CKII	D/ED/ED/ES/TD/ED/E	SFAPSDEEE	0.726
S122	DNAPK	QXS/TQE/D	EPGPSQSAS	0.575
S124	CDK1	LPXSPXKK	GPSQSASQT	0.500
S126	DNAPK	QXS/TQE/D	SQSASQTAN	0.625
T134	PKC<br>$p38^{MAPK}$	R/KR/KR/K/FSFRR<br>PXS/TP	NDTNTPKKR	0.808<br>0.518
S142	PKA	K/RK/RXS/T	RPRESSSNS	0.805
S143	PKB<br>RSK	RXRX*X*S/TF/L<br>RXRXXS/T	PRESSSNST	0.771<br>0.541
S146	CKI<br>CDK1	D/E(X)XS/T<br>LPXSPXKK	SSSNSTCTP	0.559<br>0.511
T147	PKC	R/KR/KR/K/FSFRR	SSNSTCTPP	0.605
T149	CDK5<br>$p38^{MAPK}$	S/TPXK/H/R<br>PXS/TP	NSTCTPPKR	0.645<br>0.621
S182	PKA<br>PKC	K/RK/RXS/T<br>R/KR/KR/K/FSFRR	KTLNSFVLY	0.583<br>0.513

T187	PKC	R/KR/KR/K/FSFRR	FVLYTTREK	0.872
S209	PKA<br>PKG<br>CKII	K/RK/RXS/T<br>R/KR/KXS/T<br>D/ED/ED/ES/TD/ED/E	AMFYSLHEF	0.598<br>0.544<br>0.512
S217	PKA<br>CDK1	K/RK/RXS/T<br>LPXSPXKK	FDGDSLLFG	0.645<br>0.557
S224	PKC	R/KR/KR/K/FSFRR	FLLLSGKHR	0.772
S230	PKA<br>PKG<br>CDK1	K/RK/RXS/T<br>R/KR/KXS/T<br>LPXSPXKK	KHRVSAIKN	0.653<br>0.583<br>0.570
T263	CDK5<br>p38MAPK	S/TPXK/H/R<br>PXS/TP	ALCKTPFKL	0.555<br>0.503
S271	ATM<br>DNAPK<br>PKA	L/SSQE/D<br>QXS/TQE/D<br>K/RK/RXS/T	LIKQSQEHG	0.602<br>0.574<br>0.523
T279	CKII	D/ED/ED/ES/TD/ED/E	GLSKTDFCE	0.648
S325	CKII	D/ED/ED/ES/TD/ED/E	VEGCSKCEQ	0.549
S377	PKC	R/KR/KR/K/FSFRR	LILESTREH	0.655
T378	CDK1	LPXSPXKK	ILESTREHL	0.517
T432	PKC	R/KR/KR/K/FSFRR	IKAMTENVP	0.511
T453	PKC	R/KR/KR/K/FSFRR	SGKTTVAAA	0.711
S504	DNAPK<br>CKI	QXS/TQE/D<br>D/E(X)XS/T	GSNSSLLTPG	0.562<br>0.543
T506	p38MAPK	PXS/TP	NSSLTPGMG	0.549
S512	CDK1	LPXSPXKK	GMGMSNLDN	0.525
T547	PKG	R/KR/KXS/T	PGIITMNDY	0.537
Y551	INSR	YXXM	TMNDYFIPP	0.505
T564	PKC	R/KR/KR/K/FSFRR	RMIKTINFR	0.669
S576	DNAPK	QXS/TQE/D	FLRNSLEKN	0.574
S591	PKA	K/RK/RXS/T	RIVQSGVTL	0.763
T659	DNAPK	QXS/TQE/D	ADEDTQQDS	0.501
S667	ATM<br>DNAPK<br>CKII	L/SSQE/D<br>QXS/TQE/D<br>D/ED/ED/ES/TD/ED/E	SGINSQ	0.660<br>0.649<br>0.507

STLPyV

residue	Protein kinase	Consensus motif	Sequence in LTAg	score
S6	PKC<br>CKII	R/KR/KR/K/FSFRR<br>D/ED/ED/ES/TD/ED/E	DQALSRAEA	0.659<br>0.622
S23	PKA	K/RK/RXS/T	LPEDSWGNV	0.517
S31	PKC	R/KR/KR/K/FSFRR	VPLISYRYR	0.832
S38	PKB<br>PKC<br>RSK<br>PKG	RXRX*X*S/TF/L<br>R/KR/KR/K/FSFRR<br>RXRXXS/T<br>R/KR/KXS/T	YRQKSKIYH	0.806<br>0.745<br>0.580<br>0.575
S71	CKII<br>CDK1	D/ED/ED/ES/TD/ED/E<br>LPXSPXKK	QNLRSSSEN	0.553<br>0.537
S72	PKA<br>CDK1	K/RK/RXS/T<br>LPXSPXKK	NLRSSSENE	0.615<br>0.524
S73	RSK	RXRXXS/T	LRSSSENEN	0.519
T87	p38MAPK	PXS/TP	GQYGTPAWE	0.561
T106	CKII	D/ED/ED/ES/TD/ED/E	EDDLTCNES	0.624
S114	CKII	D/ED/ED/ES/TD/ED/E	SFNCSDDEG	0.715
S122	PKC	R/KR/KR/K/FSFRR	GTSASQKRK	0.876

	DNAPK<br>CDK1	QXS/TQE/D<br>LPXSPXKK		0.573<br>0.515
S130	PKC	R/KR/KR/K/FSFRR	KFPDSSTQN	0.512
T132	DNAPK<br>PKC	QXS/TQE/D<br>R/KR/KR/K/FSFRR	PDSSTQNST	0.608<br>0.563
S135	PKC	R/KR/KR/K/FSFRR	STQNSTPPK	0.641
T136	CDK5	S/TPXK/H/R	TQNSTPPKK	0.585
S163	PKC	R/KR/KR/K/FSFRR	HAVFSNKTS	0.812
Y173	INSR	YXXM	CFCIYTTIE	0.518
T174	PKC<br>CKII<br>PKG	R/KR/KR/K/FSFRR<br>D/ED/ED/ES/TD/ED/E<br>R/KR/KXS/T	FCIYTTIEK	0.586<br>0.529<br>0.503
Y183	INSR	YXXM	GNELYTVIG	0.509
T184	PKC<br>CDK1	R/KR/KR/K/FSFRR<br>LPXSPXKK	NELYTVIGP	0.655<br>0.526
S196	PKA	K/RK/RXS/T	SMFISCHSY	0.516
S217	PKA<br>PKG	K/RK/RXS/T<br>R/KR/KXS/T	KHRVSALKN	0.596<br>0.505
S249	RSK<br>PKA	RXRXXS/T<br>K/RK/RXS/T	YRLCSAPFA	0.537<br>0.516
T255	PKC	R/KR/KR/K/FSFRR	PFAVTKQSR	0.795
S258	PKG<br>PKA	R/KR/KXS/T<br>K/RK/RXS/T	VTKQSRPEG	0.562<br>0.547
S264	DNAPK<br>CKII<br>ATM	QXS/TQE/D<br>D/ED/ED/ES/TD/ED/E<br>L/SSQE/D	PEGLSQAEF	0.586<br>0.580<br>0.575
S277	PKC	R/KR/KR/K/FSFRR	NSKPSVNWQ	0.540
S306	CKII	D/ED/ED/ES/TD/ED/E	YLDFSESPE	0.508
S308	CKI<br>CKII	D/E(X)XS/T<br>D/ED/ED/ES/TD/ED/E	DFSESPENC	0.546<br>0.527
T317	PKC	R/KR/KR/K/FSFRR	EKCKTELKH	0.546
S323	ATM	L/SSQE/D	LKHHSQFHE	0.512
Y330	EGFR<br>SRC	XXEXYYYXX<br>EEDVYGXV	HEKEYNNAK	0.543<br>0.515
S339	PKA<br>PKC	K/RK/RXS/T<br>R/KR/KR/K/FSFRR	LFRDSKTQK	0.760<br>0.664
S364	CKII	D/ED/ED/ES/TD/ED/E	LILESTRED	0.547
T365	CDK1	LPXSPXKK	ILESTREDL	0.506
S401	PKC	R/KR/KR/K/FSFRR	VAWLSLLFN	0.567
T422	CDK5	S/TPXK/H/R	MVINTPKKR	0.582
T440	PKC	R/KR/KR/K/FSFRR	SGKTTVAAA	0.711
T485	PKC	R/KR/KR/K/FSFRR	VKGQTEGKT	0.588
S502	PKC	R/KR/KR/K/FSFRR	NNLDSLRDH	0.612
S524	PKG	R/KR/KXS/T	LNKRSQIFP	0.545
T533	CKII	D/ED/ED/ES/TD/ED/E	PGIITMNEY	0.540
Y537	INSR	YXXM	TMNEYNVPL	0.506
T542	PKC	R/KR/KR/K/FSFRR	NVPLTILAR	0.516
S577	PKC<br>PKA	R/KR/KR/K/FSFRR<br>K/RK/RXS/T	RIVQSGKTL	0.693<br>0.505
S597	CKII	D/ED/ED/ES/TD/ED/E	VFHSSIHED	0.552
T608	PKC<br>PKG	R/KR/KR/K/FSFRR<br>R/KR/KXS/T	IWKETLTKY	0.711<br>0.540
T610	PKC	R/KR/KR/K/FSFRR	KETLTKYVS	0.655
T641	CKII	D/ED/ED/ES/TD/ED/E	ICEDTENNE	0.530
T646	DNAPK	QXS/TQE/D	ENNETQDSA	0.623

	CKII	D/ED/ED/ES/TD/ED/E		0.523
T653	CKII	D/ED/ED/ES/TD/ED/E	SAFCTQDSD	0.558
S656	CKII	D/ED/ED/ES/TD/ED/E	CTQDSDNE	0.655

NJPyV

residue	Protein kinase	Consensus motif	Sequence in LTAg	score
S8	CKII	D/ED/ED/ES/TD/ED/E	VLEKSDKEM	0.608
Y34	EGFR	XXEXYYYXX	MKTAYKRAS	0.531
S38	PKC<br>PKA	R/KR/KR/K/FSFRR<br>K/RK/RXS/T	YKRASKIYH	0.880<br>0.516
S57	PKC	R/KR/KR/K/FSFRR	MLLNSLWQK	0.616
S76	CKII<br>CDK1	D/ED/ED/ES/TD/ED/E<br>LPXSPXKK	SEVFSDSYG	0.559<br>0.520
Y79	INSR<br>SRC<br>EGFR	YXXM<br>EEDVYGXV<br>XXEXYYYXX	FSDSYGSAN	0.580<br>0.532<br>0.526
Y88	INSR	YXXM	FRKRYASWC	0.510
S90	PKB<br>RSK<br>PKA<br>PKC	RXRX*X*S/TF/L<br>RXRXXS/T<br>K/RK/RXS/T<br>R/KR/KR/K/FSFRR	KRYASWCSS	0.856<br>0.579<br>0.555<br>0.510
S94	PKC	R/KR/KR/K/FSFRR	SWCSSVFTN	0.596
S101	CKII	D/ED/ED/ES/TD/ED/E	TNEKSDSRA	0.532
S112	GSK3	S/TXXXS/T	HCDESPISS	0.519
S115	CKII	D/ED/ED/ES/TD/ED/E	ESPISSSSD	0.565
S116	CKII	D/ED/ED/ES/TD/ED/E	SPISSSSDE	0.665
S117	CKII	D/ED/ED/ES/TD/ED/E	PISSSSDEE	0.737
S118	CKII<br>CKI	D/ED/ED/ES/TD/ED/E<br>D/E(X)XS/T	ISSSSDEED	0.722<br>0.517
T124	CDK1	LPXSPXKK	EEDETQSSG	0.554
Y129	EGFR	XXEXYYYXX	QSSGYNSFP	0.562
T135	PKC	R/KR/KR/K/FSFRR	SFPFTSTPT	0.553
T137	CDK5<br>p38^{MAPK}	S/TPXK/H/R<br>PXS/TP	PFTSTPTPS	0.634<br>0.568
T139	CDK5<br>p38^{MAPK}	S/TPXK/H/R<br>PXS/TP	TSTPTPSTS	0.609<br>0.590
T142	PKC	R/KR/KR/K/FSFRR	PTPSTSTAS	0.751
S146	ATM<br>DNAPK	L/SSQE/D<br>QXS/TQE/D	TSTASQEVP	0.646<br>0.643
S169	PKC	R/KR/KR/K/FSFRR	SGSSSAGRN	0.724
T174	CKII	D/ED/ED/ES/TD/ED/E	AGRNTETER	0.603
T176	CKII<br>PKC	D/ED/ED/ES/TD/ED/E<br>R/KR/KR/K/FSFRR	RNTETERES	0.587<br>0.566
S180	CDK5<br>GSK3	S/TPXK/H/R<br>S/TXXXS/T	TERESPPKR	0.684<br>0.512
T188	PKA<br>RSK	K/RK/RXS/T<br>RXRXXS/T	RRRGTEDLD	0.804<br>0.558
S194	DNAPK<br>CKI	QXS/TQE/D<br>D/E(X)XS/T	DLDGSYTDS	0.566<br>0.557

	CDK1	LPXSPXKK		0.522
T196	CKII	D/ED/ED/ES/TD/ED/E	DGSYTDSQT	0.512
S198	DNAPK<br>ATM	QXS/TQE/D<br>L/SSQE/D	SYTDSQTSF	0.614<br>0.546
T200	PKC	R/KR/KR/K/FSFRR	TDSQTSFAS	0.730
S201	CKI	D/E(X)XS/T	DSQTSFAST	0.506
S204	PKC	R/KR/KR/K/FSFRR	TSFASTPPK	0.534
T205	CDK1<br>PKC	LPXSPXKK<br>R/KR/KR/K/FSFRR	SFASTPPKQ	0.737<br>0.631
S213	RSK<br>GSK3	RXRXXS/T<br>S/TXXXS/T	QKRKSPDSP	0.531<br>0.503
S216	CDK5<br>GSK3<br>p38MAPK	S/TPXK/H/R<br>S/TXXXS/T<br>PXS/TP	KSPDSPSDL	0.567<br>0.533<br>0.503
S234	PKC	R/KR/KR/K/FSFRR	HAIFSNKTV	0.793
T237	PKC	R/KR/KR/K/FSFRR	FSNKTVNAF	0.563
T246	PKC	R/KR/KR/K/FSFRR	ILYSTLEKA	0.657
S251	PKA	K/RK/RXS/T	LEKASLLYE	0.528
S266	PKA	K/RK/RXS/T	IEFKSLHKL	0.573
S292	PKA<br>PKG	K/RK/RXS/T<br>R/KR/KXS/T	KHRVSAMKN	0.650<br>0.501
S328	DNAPK	QXS/TQE/D	KPPFSQVKA	0.530
S339	CKII	D/ED/ED/ES/TD/ED/E	DGLFSYDEE	0.632
Y396	EGFR	XXEXYYYXX	KAHDYHKAH	0.555
S412	PKC	R/KR/KR/K/FSFRR	EACKSQRSI	0.651
S415	PKA	K/RK/RXS/T	KSQRSICNQ	0.513
T433	CKII	D/ED/ED/ES/TD/ED/E	RLLLTESTR	0.528
S435	CKII	D/ED/ED/ES/TD/ED/E	LLTESTREE	0.551
T510	PKC	R/KR/KR/K/FSFRR	SGKTTFAAA	0.704
S568	CDK1	LPXSPXKK	GQGISNLDN	0.550
T603	PKG<br>CKII	R/KR/KXS/T<br>D/ED/ED/ES/TD/ED/E	PCVMTMNEY	0.654<br>0.514
Y607	SRC	EEDVYGXV	TMNEYFMPQ	0.506
S618	PKA<br>PKC<br>CKI	K/RK/RXS/T<br>R/KR/KR/K/FSFRR<br>D/E(X)XS/T	FVRFSLKLD	0.759<br>0.592<br>0.511
S625	PKA	K/RK/RXS/T	LDFVSRPNL	0.553
T636	p38MAPK	PXS/TP	AVDKTPGLV	0.542
T650	PKA	K/RK/RXS/T	QKGLTLFLL	0.599
T659	CDK1<br>CDK5	LPXSPXKK<br>S/TPXK/H/R	LIWYTPVKK	0.532<br>0.500
S667	PKA<br>CKII	K/RK/RXS/T<br>D/ED/ED/ES/TD/ED/E	KFAVSLQEE	0.630<br>0.565
T703	CKII	D/ED/ED/ES/TD/ED/E	ESPLTDLID	0.604

QPyV

residue	Protein kinase	Consensus motif	Sequence in LTAg	score
S6	PKA<br>RSK<br>CKII	K/RK/RXS/T<br>RXRXXS/T<br>D/ED/ED/ES/TD/ED/E	DRLLSRDEV	0.587<br>0.580<br>0.578
S20	PKA<br>CDK1	K/RK/RXS/T<br>LPXSPXKK	LIGLSMSNW	0.718<br>0.577
S22	CDK1	LPXSPXKK	GLSMSNWGN	0.531
T65	PKC	R/KR/KR/K/FSFRR	KFAATMRDQ	0.835

S70	PKA	K/RK/RXS/T	MRDQSSGNP	0.764
S79	CKII	D/ED/ED/ES/TD/ED/E	IWHFSSEEV	0.533
T91	CDK5<br>$p38^{MAPK}$<br>GSK3	S/TPXK/H/R<br>PXS/TP<br>S/TXXXS/T	PPYGTPEWD	0.583<br>0.548<br>0.500
Y110	SRC	EEDVYGXV	DEDLYCTEE	0.539
S116	CKII	D/ED/ED/ES/TD/ED/E	TEELSASDE	0.622
S118	CKII<br>CDK1	D/ED/ED/ES/TD/ED/E<br>LPXSPXKK	ELSASDEEQ	0.683<br>0.524
T123	CKII	D/ED/ED/ES/TD/ED/E	DEEQTAEDP	0.635
S132	ATM<br>DNAPK<br>CDK1	L/SSQE/D<br>QXS/TQE/D<br>LPXSPXKK	EEGCSQNSK	0.565<br>0.536<br>0.519
Y137	EGFR	XXEXYYYXX	QNSKYSATP	0.514
S138	PKG	R/KR/KXS/T	NSKYSATPP	0.520
T140	CDK5<br>$p38^{MAPK}$	S/TPXK/H/R<br>PXS/TP	KYSATPPKQ	0.593<br>0.543
T179	PKC<br>CKII	R/KR/KR/K/FSFRR<br>D/ED/ED/ES/TD/ED/E	FVCYTTMEK	0.532<br>0.502
S184	PKA	K/RK/RXS/T	TMEKSLMLY	0.546
S209	PKA	K/RK/RXS/T	CNDGSGSIV	0.592
S219	PKC<br>CDK1	R/KR/KR/K/FSFRR<br>LPXSPXKK	MITGSRHRP	0.795<br>0.581
S224	PKA<br>PKG	K/RK/RXS/T<br>R/KR/KXS/T	RHRPSAILN	0.719<br>0.532
S230	PKC<br>CDK1	R/KR/KR/K/FSFRR<br>LPXSPXKK	ILNASKKYC	0.870<br>0.534
T235	PKC	R/KR/KR/K/FSFRR	KKYCTVSFS	0.631
S237	PKC	R/KR/KR/K/FSFRR	YCTVSFSLV	0.598
T260	PKC	R/KR/KR/K/FSFRR	GPNFTVIRE	0.591
S265	PKA	K/RK/RXS/T	VIRESREGG	0.572
S272	CDK1<br>CKII	LPXSPXKK<br>D/ED/ED/ES/TD/ED/E	GGLHSYDFQ	0.575<br>0.549
S279	PKC	R/KR/KR/K/FSFRR	FQEASKKFF	0.518
S343	PKG	R/KR/KXS/T	KLFKSAKVNQ	0.520
T352	DNAPK	QXS/TQE/D	RGIATQAAD	0.635
S360	PKC<br>RSK	R/KR/KR/K/FSFRR<br>RXRXXS/T	DRVLSARRV	0.673<br>0.573
S369	PKC	R/KR/KR/K/FSFRR	MMIESTRVD	0.579
T414	PKC	R/KR/KR/K/FSFRR	MPSFTTKLK	0.748
T415	PKC	R/KR/KR/K/FSFRR	PSFTTKLKE	0.676
T445	PKC	R/KR/KR/K/FSFRR	NSGKTTVAA	0.555
T446	PKC	R/KR/KR/K/FSFRR	SGKTTVAAG	0.529
S466	PKC	R/KR/KR/K/FSFRR	VNCSSDKIN	0.611
T526	PKC<br>PKG	R/KR/KR/K/FSFRR<br>R/KR/KXS/T	ERKHTNKVS	0.751<br>0.530
S530	CDK1	LPXSPXKK	TNKVSQLFP	0.512
T539	PKG	R/KR/KXS/T	PCIITCNDY	0.516
Y543	INSR	YXXM	TCNDYAIPR	0.545
T548	PKC	R/KR/KR/K/FSFRR	AIPRTVKAR	0.851
S598	CKII	D/ED/ED/ES/TD/ED/E	WEPVSEFVE	0.628
Y621	SRC	EEDVYGXV	RWVTYGMYQ	0.507
Y639	SRC	EEDVYGXV	KDPFYGVIM	0.553
S644	CKII<br>PKA	D/ED/ED/ES/TD/ED/E<br>K/RK/RXS/T	GVIMSDINE	0.577<br>0.534

T653	DNAPK<br>ATM	QXS/TQE/D<br>L/SSQE/D	IVEETQEST	0.603<br>0.505
S656	CKI	D/E(X)XS/T	ETQESTESG	0.517
T657	CKI	D/E(X)XS/T	TQESTESGV	0.575
S659	CKI	D/E(X)XS/T	ESTESGVGS	0.597
S663	CKI<br>DNAPK<br>CKII	D/E(X)XS/T<br>QXS/TQE/D<br>D/ED/ED/ES/TD/ED/E	SGVGSMET	0.536<br>0.525<br>0.525

References

1. Blom, N.; Sicheritz-Pontén, T.; Gupta, R.; Gammeltoft, S.; Brunak S. Proteomics Prediction of post-translational glycosylation and phosphorylation of proteins from the amino acid sequence. *Proteomic* **2004**, *4*,1633-1649.
2. Blom, N.; Gammeltoft, S.; Brunak S. Sequence and structure-based prediction of eukaryotic protein phosphorylation sites. *J. Mol. Biol.* **1999**, *294*, 1351-1362.
3. Rust, H.L.; Thompson, P.R. Kinase consensus sequences: a breeding ground for crosstalk. *ACS Chem. Biol.* **2011,** *6,* 881-892.
4. Safaei, J.; Maňuch, J.; Gupta, A.; Stacho, L.; Pelech, S. Prediction of 492 human protein kinase substrate specificities. *Proteome Sci.* **2011**, 9 *Suppl 1,* S6.
5. Copps, K.D., White, M.F. Regulation of insulin sensitivity by serine/threonine phosphorylation of insulin receptor substrate proteins IRS1 and IRS2. *Diabetologia* **2012,** *55,* 2565-2582.
6. Hornbeck, P.V.; Zhang, B.; Murray, B.; Kornhauser, J.M.; Latham, V.; Skrzypek E. PhosphoSitePlus 2014: mutations, PTMs and recalibrations. Nucleic Acids Res. **2015,** *43,* D512-520.
7. Begley, M.J.; Yun, C.H.; Gewinner, C.A.; Asara, J.M.; Johnson, J.L.; Coyle, A.J.; Eck, M.J.; Apostolou, I.; Cantley, L.C. EGF-receptor specificity for phosphotyrosine-primed substrates provides signal integration with Src. *Nat. Struct. Mol. Biol.* **2015,** *22,* 983-990.

Supplementary Table S2. Putative phosphorylation sites in SV40 sTAg and human polyomaviruses and their protein kinase. The algorithm NetPhos3.1 was used to predict protein kinases [1,2]. The protein kinase consensus motifs are based on [3-7].

SV40

residue	Protein kinase	Consensus motif	Sequence in sTAg	score
S10	PKA	K/RK/RXS/T	REES	0.749
S22	CKI<br>CDK1	D/E(X)XS/T<br>LPXSPXKK	ERS<br>GLERSAWGN	0.510<br>0.503
T57	PKC	R/KR/KR/K/FSFRR	KKMNT	0.842
T81	CDK1	LPXSPXKK	FWDATEVFA	0.511
S87	CKI	PS/GS/TPXR/K	VFASSLNPG	0.509
Y96	EGFR	XXEXYYYXX	VDAMYCKQW	0.532
S108	PKA	K/RK/RXS/T	KKMS	0.653
T167	PKC	R/KR/KR/K/FSFRR	IIGQTTYR	0.755
T168	PKC	R/KR/KR/K/FSFRR	IIGQTTYR	0.654

BKPyV

residue	Protein kinase	Consensus motif	Sequence in sTAg	score
T57	PKC	R/KR/KR/K/FSFRR	KRMNTLYKK	0.798
S78	CKII	D/ED/ED/ES/TD/ED/E	FGTWSSSEV	0.584
S79	CDK1	LPXSPXKK	FGTWSSSEV	0.506
S80	CDKI<br>CKII	LPXSPXKK<br>D/ED/ED/ES/TD/ED/E	FGTWSSSEV<br>FGTWSSSEV	0.525<br>0.518
S102	PKC	R/KR/KR/K/FSFRR	WPICSKKPS	0.727
S106	PKC<br>PKA	R/KR/KR/K/FSFRR<br>K/RK/RXS/T	SKKPSVHCP<br>SKKPSVHCP	0.603<br>0.534
T165	p38MAPK	PXS/TP	IGETPFRD	0.556

JCPyV

residue	Protein kinase	Consensus motif	Sequence in sTAg	score
S79	CDK1	LPXSPXKK	GTWNSSEVG	0.506
T103	PKC	R/KR/KR/K/FSFRR	PNCATNPSV	0.512
S128	PKA	K/RK/RXS/T	KFLRSSPLV	0.545
S129	PKA<br>CDK1	K/RK/RXS/T<br>LPXSPXKK	KFLRSSPLV<br>KFLRSSPLV	0.596<br>0.503
T151	DNAPK<br>ATM	QXS/TQE/D<br>L/SSQE/D	GCDLTQEAL	0.617<br>0.564

KIPyV

residue	Protein kinase	Consensus motif	Sequence in sTAg	score
S6	CKII	D/ED/ED/ES/TD/ED/E	DKTLSREEA	0.518
S65	CDK1	LPXSPXKK	KLQDSVSSV	0.514
S83	DNAPK<br>CDK1	QXS/TQE/D<br>LPXSPXKK	IWQSSQVYC	0.591<br>0.520
Y102	EGFR	XXEXYYYXX	VNAIYGEYY	0.643
Y105	EGFR<br>SRC	XXEXYYYXX<br>EEDVYGXV	IYGEYYEAY	0.527<br>0.508
Y106	SRC<br>INSR	EEDVYGXV<br>YXXM	YGEYYEAYI	0.529<br>0.523
Y109	EGFR	XXEXYYYXX	YYEAYIMKQ	0.543

S133	PKC	R/KR/KR/K/FSFRR	HCILSKYHK	0.755
T170	PKC	R/KR/KR/K/FSFRR	ISMQTFFFW	0.722

WUPyV

residue	Protein kinase	Consensus motif	Sequence in sTAg	score
T4	CDK1	LPXSPXKK	MDKTLSRN	0.505
T32	PKC	R/KR/KR/K/FSFRR	PLMRTKYLS	0.512
S36	PKC	R/KR/KR/K/FSFRR	TKYLSKCKE	0.638
S57	PKA<br>PKC	K/RK/RXS/T<br>R/KR/KR/K/FSFRR	KKLNSLYLK	0.681<br>0.620
S81	CKII	D/ED/ED/ES/TD/ED/E	DEVWSSSQV	0.566
S82	CKII	D/ED/ED/ES/TD/ED/E	DEVWSSSQV	0.544
S83	CKII<br>DNAPK<br>CDK1<br>CKI	D/ED/ED/ES/TD/ED/E<br>QXS/TQE/D<br>LPXSPXKK<br>PS/GS/TPXR/K	DEVWSSSQV	0.618<br>0.603<br>0.531<br>0.530
Y106	EGFR<br>SRC	XXEXYYYXX<br>EEDVYGXV	VGEVYGDVF	0.507<br>0.507
T174	PKC<br>CKI	R/KR/KR/K/FSFRR<br>PS/GS/TPXR/K	ISAETFFYW	0.767<br>0.550
T185	PKC	R/KR/KR/K/FSFRR	IIFLTTMQG	0.545

MCPyV

residue	Protein kinase	Consensus motif	Sequence in sTAg	score
Y24	INSR	YXXM	APNCYGNIP	0.568
S35	PKC<br>PKG	R/KR/KR/K/FSFRR<br>R/KR/KXS/T	AFKRSCLKH	0.567<br>0.521
T57	PKC	R/KR/KR/K/FSFRR	MELNTLWSK	0.571
S60	PKC	R/KR/KR/K/FSFRR	NTLWSKFQQ	0.654
S80	PKC<br>CKI	R/KR/KR/K/FSFRR<br>PS/GS/TPXR/K	FDEVSTKFP	0.608<br>0.538
T81	CKII	D/ED/ED/ES/TD/ED/E	DEVSTKFPW	0.563
T90	PKC	R/KR/KR/K/FSFRR	EEGTLKDY	0.677
S117	PKA	K/RK/RXS/T	QLRDSKCAC	0.592
S123	PKC	R/KR/KR/K/FSFRR	CACISCKLS	0.635
S132	PKC	R/KR/KR/K/FSFRR	RQHCSLKTL	0.657
T135	PKC	R/KR/KR/K/FSFRR	CSLKTLKQK	0.922
T143	PKG	R/KR/KXS/T	KNCLTWGEC	0.501
T172	CKII	D/ED/ED/ES/TD/ED/E	WWQKTLEET	0.589
Y178	EGFR	XXEXYYYXX	EETDYCLLH	0.514

HPyV6

residue	Protein kinase	Consensus motif	Sequence in sTAg	score
S20	PKA<br>CDK1	K/RK/RXS/T<br>LPXSPXKK	LIGLSMACW	0.631<br>0.532
T65	PKC	R/KR/KR/K/FSFRR	KLNATLRDQ	0.572
S73	ATM	L/SSQE/D	QMSSSPTWC	0.520
T75	PKC	R/KR/KR/K/FSFRR	SSSPTWCFS	0.661

S80	CKII	D/ED/ED/ES/TD/ED/E	WCFSSEVSD	0.505
S83	CKII	D/ED/ED/ES/TD/ED/E	SSEVSDDWG	0.597
T91	PKC	R/KR/KR/K/FSFRR	GIPLTVGEF	0.727
S117	CDK1	LPXSPXKK	QQGISSCKC	0.518
S118	PKC	R/KR/KR/K/FSFRR	GGISSCKCL	0.671
S176	CDK5	S/TPXK/H/R	IIYQSPLDW	0.509

HPyV7

residue	Protein kinase	Consensus motif	Sequence in sTAg	score
S38	PKC<br>PKA	R/KR/KR/K/FSFRR<br>K/RK/RXS/T	VRLASKKYH	0.921<br>0.508
S73	RSK<br>$p38^{MAPK}$	RXRXXS/T<br>PXS/TP	QRSGSPMWH	0.537<br>0.500
S79	CKII	D/ED/ED/ES/TD/ED/E	MWHYSSDEV	0.585
S80	CKII	D/ED/ED/ES/TD/ED/E	WHYSSDEVS	0.534
S84	CKII	D/ED/ED/ES/TD/ED/E	SDEVSFWDI	0.530
T91	CKII	D/ED/ED/ES/TD/ED/E	DIELTVGEF	0.527
S168	CKI	PS/GS/TPXR/K	RSEESFMWW	0.536
S173	PKA	K/RK/RXS/T	FMWWSHIIF	0.515
T179	$p38^{MAPK}$	PXS/TP	IIFQTPMDV	0.517

TSPyV

residue	Protein kinase	Consensus motif	Sequence in sTAg	score
S6	CKII<br>CKI	D/ED/ED/ES/TD/ED/E<br>PS/GS/TPXR/K	DKFLSREES	0.598<br>0.517
S10	PKA<br>DNAPK	K/RK/RXS/T<br>QXS/TQE/D	SREESLELM	0.660<br>0.503
S38	PKC<br>PKA	R/KR/KR/K/FSFRR<br>K/RK/RXS/T	HKKMSLKYH	0.921<br>0.565
S53	PKA	K/RK/RXS/T	PEKMSRLNQ	0.611
Y67	EGFR<br>SRC	XXEXYYYXX<br>EEDVYGXV	QEGIYNARQ	0.629<br>0.513
S76	CDK1	LPXSPXKK	EFPTSFSSQ	0.560
S78	CDK1	LPXSPXKK	PTSFSSQVG	0.515
S79	ATM<br>DNAPK	L/SSQE/D<br>QXS/TQE/D	TSFSSQVGS	0.673<br>0.572
S83	CKII<br>CDK1	D/ED/ED/ES/TD/ED/E<br>LPXSPXKK	SQVGSWYWE	0.511<br>0.508
Y85	EGFR	XXEXYYYXX	VGSWYWEAN	0.501
S92	CKI<br>PKG	PS/GS/TPXR/K<br>R/KR/KXS/T	ANLISLKEY	0.546<br>0.528
T125	PKC	R/KR/KR/K/FSFRR	CKCLTCKIG	0.589
S173	CDK1	LPXSPXKK	LWAWSCIVG	0.563

HPyV9

residue	Protein kinase	Consensus motif	Sequence in sTAg	score
S6	CKII<br>DNAPK	D/ED/ED/ES/TD/ED/E<br>QXS/TQE/D	DQTLSLEER	0.622<br>0.505
S28	PKC	R/KR/KR/K/FSFRR	WGNLSLMKK	0.521
Y34	EGFR	XXEXYYYXX	MKKAYKTVS	0.515
T36	PKC	R/KR/KR/K/FSFRR	KAYKTVSKI	0.831

S38	PKC	R/KR/KR/K/FSFRR	YKTVSKIYH	0.873
S76	CDK1	LPXSPXKK	NCGSSSSQV	0.548
S78	DNAPK<br>ATM	QXS/TQE/D<br>L/SSQE/D	GSSSSQVAW	0.603<br>0.529
T113	PKC	R/KR/KR/K/FSFRR	PDCITYNKP	0.727
Y114	EGFR	XXEXYYYXX	DCITYNKPS	0.502
S118	PKA	K/RK/RXS/T	YNKPSCCCI	0.603
S132	PKC	R/KR/KR/K/FSFRR	QQHKSTKIN	0.898
T133	PKC<br>PKG	R/KR/KR/K/FSFRR<br>R/KR/KXS/T	QHKSTKINK	0.861<br>0.547
S179	PKA	K/RK/RXS/T	NMDLSLLRL	0.579

HPyV10

residue	Protein kinase	Consensus motif	Sequence in sTAg	score
S6	CKII<br>RSK<br>CKI	D/ED/ED/ES/TD/ED/E<br>RXRXXS/T<br>PS/GS/TPXR/K	DRVLSRDEV	0.576<br>0.540<br>0.502
T37	PKC	R/KR/KR/K/FSFRR	KYRQTCLKL	0.714
S60	PKC	R/KR/KR/K/FSFRR	NELFSKMYT	0.531
Y63	INSR	YXXM	FSKMYTTIE	0.522
T64	PKC	R/KR/KR/K/FSFRR	SKMYTTIEK	0.639
Y76	SRC	EEDVYGXV	EGEVYFPAK	0.518
T90	CKI	PS/GS/TPXR/K	DDVVTLGDV	0.520
S98	CKII	D/ED/ED/ES/TD/ED/E	VLGPSFEEK	0.514
Y105	EGFR<br>INSR	XXEXYYYXX<br>YXXM	EKIIYIWPL	0.611<br>0.517
Y163	INSR	YXXM	QEFGYTSFF	0.521
S195	PKC<br>CKI	R/KR/KR/K/FSFRR<br>PS/GS/TPXR/K	VSYFSFILG	0.638<br>0.512

STLPyV

residue	Protein kinase	Consensus motif	Sequence in sTAg	score
S6	PKC<br>CKII	R/KR/KR/K/FSFRR<br>D/ED/ED/ES/TD/ED/E	DQALSRAEA	0.659<br>0.622
S23	PKA	K/RK/RXS/T	LPEDSWGNV	0.517
S31	PKC	R/KR/KR/K/FSFRR	VPLISYRYR	0.832
S38	PKB<br>PKC<br>RSK<br>PKG	RXRX*X*S/TF/L<br>R/KR/KR/K/FSFRR<br>RXRXXS/T<br>R/KR/KXS/T	YRQKSKIYH	0.806<br>0.745<br>0.580<br>0.575
S71	CKII<br>CDK1	D/ED/ED/ES/TD/ED/E<br>LPXSPXKK	QNRSSSEN	0.553<br>0.537
S72	PKA<br>CDK1	K/RK/RXS/T<br>LPXSPXKK	NLRSSSENE	0.615<br>0.524
S73	RSK	RXRXXS/T	LRSSSENEN	0.519
T90	CDK1	LPXSPXKK	LLSDTFTLG	0.522
T92	CKI	PS/GS/TPXR/K	SDTFTLGDL	0.592
S103	CDK1	LPXSPXKK	PQFESKVIF	0.536
T111	PKC	R/KR/KR/K/FSFRR	FIWPTCAKC	0.502
T119	PKG	R/KR/KXS/T	CRYRTFCKC	0.645
T145	PKG	R/KR/KXS/T	KPCVTWGEC	0.538
S167	PKC<br>PKA	R/KR/KR/K/FSFRR<br>K/RK/RXS/T	FTKTSLHAW	0.722<br>0.525

X*=polar amino acid

NJPyV

residue	Protein kinase	Consensus motif	Sequence in sTAg	score
S8	CKII	D/ED/ED/ES/TD/ED/E	VLEKSDKEM	0.608
Y34	EGFR	XXEXYYYXX	MKTAYKRAS	0.531
S38	PKC<br>PKA	R/KR/KR/K/FSFRR<br>K/RK/RXS/T	YKRASKIYH	0.880<br>0.518
S57	PKC	R/KR/KR/K/FSFRR	MLLNSLWQK	0.616
S78	CDK1	LPXSPXKK	VCQVSFSDC	0.551
S80	CDK1	LPXSPXKK	QVSFSDCYD	0.543
S85	CDK1	LPXSPXKK	DCYDSSLLK	0.520
S86	PKC	R/KR/KR/K/FSFRR	CYDSSLLKC	0.506
S92	p38 MAPK<br>CDK1	PXS/TP<br>LPXSPXKK	LKCCSPKVF	0.509<br>0.505
S103	p38 MAPK<br>CDK5	PXS/TP<br>S/TPXK/H/R	LFLRSPQCL	0.584<br>0.526
T138	PKA<br>PKG<br>PKC	K/RK/RXS/T<br>R/KR/KXS/T<br>R/KR/KR/K/FSFRR	KRCLTWGNC	0.786<br>0.538<br>0.524
T157	PKC	R/KR/KR/K/FSFRR	GLRETWKTF	0.692

QPyV

residue	Protein kinase	Consensus motif	Sequence in sTAg	score
S6	PKA<br>RSK<br>CKII	K/RK/RXS/T<br>RXRXXS/T<br>D/ED/ED/ES/TD/ED/E	DRLLSRDEV	0.587<br>0.580<br>0.578
S20	PKA<br>CDK1	K/RK/RXS/T<br>LPXSPXKK	LIGLSMSNW	0.718<br>0.577
S22	CDK1	LPXSPXKK	GLSMSNWGN	0.531
T65	PKC	R/KR/KR/K/FSFRR	KFAATMRDQ	0.835
S70	PKA	K/RK/RXS/T	MRDQSSGNP	0.764
S79	CKII	D/ED/ED/ES/TD/ED/E	IWHFSSEEV	0.547
S80	CKII	D/ED/ED/ES/TD/ED/E	WHFSSEEVS	0.500
T173	DNAPK	QXS/TQE/D	FEWWTQITY	0.561
T179	DNAPK<br>ATM	QXS/TQE/D<br>L/SSQE/D	IIYGTQMDV	0.549<br>0.501

References

1. Blom, N.; Sicheritz-Pontén, T.; Gupta, R.; Gammeltoft, S.; Brunak S. Proteomics Prediction of post-translational glycosylation and phosphorylation of proteins from the amino acid sequence. *Proteomic* **2004**, *4*,1633-1649.
2. Blom, N.; Gammeltoft, S.; Brunak S. Sequence and structure-based prediction of eukaryotic protein phosphorylation sites. *J. Mol. Biol.* **1999**, *294*, 1351-1362.
3. Rust, H.L.; Thompson, P.R. Kinase consensus sequences: a breeding ground for crosstalk. *ACS Chem. Biol.* **2011,** *6,* 881-892.
4. Safaei, J.; Maňuch, J.; Gupta, A.; Stacho, L.; Pelech, S. Prediction of 492 human protein kinase substrate specificities. *Proteome Sci.* **2011**, 9 *Suppl 1,* S6.
5. Copps, K.D., White, M.F. Regulation of insulin sensitivity by serine/threonine phosphorylation of insulin receptor substrate proteins IRS1 and IRS2. *Diabetologia* **2012,** *55,* 2565-2582.
6. Hornbeck, P.V.; Zhang, B.; Murray, B.; Kornhauser, J.M.; Latham, V.; Skrzypek E. PhosphoSitePlus 2014: mutations, PTMs and recalibrations. Nucleic Acids Res. **2015,** *43,* D512-520.

7. Begley, M.J.; Yun, C.H.; Gewinner, C.A.; Asara, J.M.; Johnson, J.L.; Coyle, A.J.; Eck, M.J.; Apostolou, I.; Cantley, L.C. EGF-receptor specificity for phosphotyrosine-primed substrates provides signal integration with Src. *Nat. Struct. Mol. Biol.* **2015,** *22,* 983-990.

Supplementary Figure S1. Alignment of LTAg from SV40 and HPyVs. The known phosphorylation sites in SV40 are shown in grey and conserved residues are highlighted in green. Clustal was used for alignment [1].

Alphaviruses

```
SV      MDKVLNREESLQLMDLLGLERSAWGNIPLMRKAYLKKCKEFHPDKGGDEEKMKKMNTLYK     60
TS      MDKFLSREESLELMDLLQIPRHCYGNFALMKINHKKMSLKYHPDKGGDPEKMSRLNQLWQ     60
H9      MDQTLSLEERNELMDLLQLTRAAWGNLSLMKKAYKTVSKIYHPDKGGNPEKMQRLNELFQ     60
MC      MDLVLNRKEREALCKLLEIAPNCYGNIPLMKAAFKRSCLKHHPDKGGNPVIMMELNTLWS     60
NJ      MEKVLEKSDKEMLIELLGIPRYAYGNFPIMKTAYKRASKIYHPDKGGSSEKMMLLNSLWQ     60
        *:  *. .:   * .** :   .:**: :*:  .   .  .******.   *  :* *:.

SV      KMEDGVKYAHQPDFGG------------------FWDATEIPTYGTDEWEQW---WNAFN     99
TS      KLQEGIYNARQE-FPTSFSSQH--DVPT-------QDGRDIPPYGHPSWASW---WESFN     107
H9      KLQVTLLEIRSN-CGSSSSQGYYSDSPYFTETPFSYCERKNEDPEGGSWGKW---WREFV     116
MC      KFQQNIHKLRSD-FSMFDEVD---EA---------------PIYGTTKFKEW---WRSGG     98
NJ      KFQEGLIEVRDS-EVFSD------------------------SYGSANFRKRYASWCSSV     95
        *::  :   :.                                    .: .    *

SV      E-----------------------------------------------------------     100
TS      QEWD--------------------------------------------------------     111
H9      NK----------------------------------------------------------     118
MC      FSFGKAYEYGPNPHGTNSRSRKPSSNASRGAPSGSSPPHSQSSSSGYGSFSASQASDSQS     158
NJ      FTNEKSD----------SRA---DLHCDESPISSSSDEEDETQSSGYNSFPFTSTP----     138


SV      ---------------------------------------------------ENLFCSEEM     109
TS      -------------------------------------------NLFDTMQDPDLFCHEST     128
H9      -------------------------------------------------EYDDLFCSETI     129
MC      RGPDIPPEHHEEPTSSSGSSSREETTNSGRESSTPNGTSVPRNSSRTDGTWEDLFCDESL     218
NJ      ------------------------------------------------------------     138


SV      PSSDDEAT----------------------------------------------------     117
TS      IPSDESR------SP-----SPTPGPSTQFSEENSRR-------------RRAAPP----     160
H9      SSSDDEN------NP-----GPSAPPPSSAS-----A-------------SEDPDP----     156
MC      SSPEPPSSSEEPEEPPSSRSSPRQPPSSSAEEASSSQFTDEEYRSSSFTTPKTPPPFSRK     278
NJ      ---TP-STSTASQEVPPPFSEPQFPESSSASGSSSAGRNT--------ETERESPPKRRR     186


SV      --------------ADSQHSTPPKKK--RKVEDPKDFPSELLSFLSHAVFSNRTLACFAI     161
TS      -------EDSPGCTQSSFSATPPKPKKSKYDSVPNDFPDMLRPFLSNAVYSNKTLSSFLI     213
H9      -------EEEAGSSQSSFTCTPPKRKKPE-PNTPEDFPMCLYSFLSHAIYSNKTMNCFLI     208
MC      RKFGGSRSSASSASSASFTSTPPKPKKNRETPVPTDFPIDLSDYLSHAVYSNKTVSCFAI     338
NJ      G--TEDLDGSYTDSQTSFASTPPKQKR-KSPDSPSDLPSCLFDFVSHAIFSNKTVNAFIL     243
                        *  .**** *  .    * *:*  *  ::*:*::**:*: .* :

SV      YTTKEKAALLYKKIMEK-----YSVTFISRHNSYN-----HNILFFLTPHRHRVSAINNY     211
TS      YTTNEKAEYLYKKLDKFN------PEFKSRHSFQE-----GSMVFLMTPGKHRVSAIKNL     262
H9      YTTVEKSKQLYRTVEKSKIKVDFKAIFLYKDDGIE-----GGLLYFITLGKHRVSAVKHF     263
MC      YTTSDKAIELYDKIEKFKV------DFKSRHACEL-----GCILLFITLSKHRVSAIKNF     387
NJ      YSTLEKASLLYEKIDKFKI------EFKSLHKLTEGANVGGGLVLVMTIAKHRVSAMKNF     297
        *:* :*:  ** .: :          *   .           :: .:*  :*****:::

SV      AQKLCTFSFLICKGVNKEYLMYSALTRDPFSVIEESLPGGLKEHDFNPEEAEETKQVSWK     271
TS      CVTHCTVSFLLCKAVIKQVECYRCMCSEPFKLLEESKPGIF-EYEF--NEENGKPVVNWN     319
H9      CVAQCTFSFIHCKAVIKPLELYRALGKPPFKLLEENKPGVS-MFDF--QEEKE-QAVNWQ     319
MC      CSTFCTISFLICKGVNKMPEMYNNLCKPPYKLLQENKP-LL-NYEF--QEKEKEASCNWN     443
NJ      CQQFCTVSFLICKVVLKPLECYQCLCKPPFSQVKANKDGLF-SYDF--ED-KKEENCNWN     353
        .   **.**: ** * *    *  :   *:. :: .       .:*  :: :     .*:

SV      LVTEYAMETKCDDVLLLLGMYLEFQYSFEMCLKCIKKEQPSHYKYHEKHYANAAIFADSK     331
TS      LLTDFAVTNRLDDPLLIMAHYLDFAEEPSICSKCTKKALKAHYNYHSLHHKNAKLFKECK     379
H9      EICNYAVEAKITDVLLLLGIYLDFAVEPGTCSKCEKKSHKFHYNYHSKHHANACLFLESK     379
MC      LVAEFACEYELDDHFIILAHYLDFA-KPFPCQKCENRSRLKPHKAHEAHHSNAKLFYESK     502
```

```
NJ      KVAEFAVLADIDDPLLILAHYLDFA-QPFPCLKCEHQK-TKAHDYHKAHHENAVLFEACK        411
         : ::*      * ::::. **:*  .    * ** ::      :. *. *: ** :*  .*

SV      NQKTICQQAVDTVLAKKRVDSLQLTREQMLTNRFNDLLDRMDIMFGSTGSADIEEWMAGV        391
TS      TQKTACQQAADVVMAKQRLKLIESTRKELLEERFKLMFEKLTDE---FGQIKILQYMAGV        436
H9      SQKNICQQAVDQVLAAKRLKLVECTRMELLEDRFIQLFDEMEDFL--HGEIEILRWMSGV        437
MC      SQKTICQQAADTVLAKRRLEMLEMTRTEMLCKKFKKHLERLRDL----DTIDLLYYMGGV        558
NJ      SQRSICNQASDIVLAKRRLLLTESTREELLAMCFQKQLKALQAL----DTLEIYDHMAGV        467
        .*:. *:** * *:* :*:   : ** ::*   *    :. :        .  .:   *.**

SV      AWLHCLLPKMDSVVYDFLKCMVYNIPKKRYWLFKGPIDSGKTTLAAALLELCGGKALNVN        451
TS      AWYSCLFENIDEVVTKILKLIVENVPKKRNCLFRGPINSGKTTFAAALMNFLGGKTLNVN        496
H9      AWYTILLDNSWDVFQKILQLVTTSQPKKRNILFKGPINSGKTTLASAFMHFFDGKALNIN        497
MC      AWYCCLFEEFEKKLQKIIQLLTENIPKYRNIWFKGPINSGKTSFAAALIDLLEGKALNIN        618
NJ      AWYANLFENFDDILFQILKLLTQNIPKQRNILFRGPVNSGKTTFAAALVDLLGGRSLNVN        527
        **   *: :  . . .::: :. . ** *   *:**::****::*:*::.:  *::**:*

SV      LPLDRLNFELGVAIDQFLVVFEDVKGTGGESRDLPSGQGINNLDNLRDYLDGSVKVNLEK        511
TS      CPADKLPFELGCAIDQFVVIFEDVKGQIALNKKLQPGQGVSNLDNLRDHLDGSVKVDLER        556
H9      CPAEKLSFELGCAIDQFCVLLDDVKGQITLNKHLQPGQGVNNLDNLRDHLDGTIKVNLEK        557
MC      CPSDKLPFELGCALDKFMVVFEDVKGQNSLNKDLQPGQGINNLDNLRDHLDGAVAVSLEK        678
NJ      CPADKLNFELGCAIDRFFVVFEDVKGQNMLNKKLQPGQGISNLDNMRDYLDGAVPVNLEK        587
         * ::* **** *:*:* *:::****     .:.*  ***:.****:**:***:: *.**:

SV      KHLNKRTQIFPPGIVTMNEYSVPKTLQARFVKQIDFRPKDYLKHCLERSEFLLEKRIIQS        571
TS      KHVNKRSQIFPPCLVTMNEYLLPETIFTRFAYVLNFTPKHNLRSCLQVSDYLLTERILQD        616
H9      KHVNKRSQIFPPVIMTMNEYLLPPTVGVRFALHIHFHCKTYLKQSLEKSD-LIEKRILNS        616
MC      KHVNKKHQIFPPCIVTANDYFIPKTLIARFSYTLHFSPKANLRDSLDQNMEIRKRRILQS        738
NJ      KHMNKRSQVFPPCVMTMNEYFMPQTLFVRFSLKLDFVSRPNLQSAVDKTPGLVANRILQK        647
        **:**: *:*** ::* *:* :* *: .**   :.*  :  *: .:: .  :  .**::.

SV      GIALLLMLIWYRPVAEFAQSIQSRIVEWKERLDKEFSLSVYQKMKFNVAMGIGVLDWLRN        631
TS      GVTIALLLVWYCPITMFSESIKEDVKYWKDILCKYMGHTNFATLLLNVEEGKDPLDSVVI        676
H9      GYTILLLLLWYNPVDSFTPKVQEYVVKWKEILERHVSITQFGNIQQNILDGKDPLHGIVI        676
MC      GTTLLLCLIWCLPDTTFKPCLQEEIKNWKQILQSEISYGKFCQMIENVEAGQDPLLNILI        798
NJ      GLTLFLLLIWYTPVKKFAVSLQEEIANWKCIIEKTVSHSDFCKMLENIEVGESPLTDLID        707
        * :: * *:*  *   *   ::. :  **  :   ..   :  :  *:  * . *  :

SV      SDDDDEDSQENADKNEDGGEKNMEDSGHETGIDSQSQGSFQAPQSSQSVHDHNQPYHICR        691
TS      EVEDEEEEF-SET---------NDSGFQ----TQ-------------------------        697
H9      EEQA--------------------------------------------------------        680
MC      EEEGPEETE---ET---------QDSGTF----SQ-------------------------        817
NJ      EGDN--------------------------------------------------------        711
        . :

SV      GFTCFKKPPTPPPEPET 708
TS      ----------------- 697
H9      ----------------- 680
MC      ----------------- 817
NJ      ----------------- 711
```

Betapolyomaviruses

```
SV      MDKVLNREESLQLMDLLGLERSAWGNIPLMRKAYLKKCKEFHPDKGGDEEKMKKMNTLYK        60
BK      MDKVLNREESMELMDLLGLERAAWGNLPLMRKAYLRKCKEFHPDKGGDEDKMKRMNTLYK        60
JC      MDKVLNREESMELMDLLGLDRSAWGNIPVMRKAYLKKCKELHPDKGGDEDKMKRMNFLYK        60
KI      MDKTLSREEAKQLMQLLCLDMSCWGNLPLMRRQYLVKCKEYHPDKGGNEESMKLLNSLYL        60
WU      MDKTLSRNEAKELMQLLGLDMTCWGNLPLMRTKYLSKCKEFHPDKGGNEEKMKKLNSLYL        60
        ***.*.*:*: :**:** *: :.***:*:**  ** **** ******:*:.** :* **

SV      KMEDGVKYAHQPD--FGGFWDATEIPTYGTDEWEQWWNAFNE---ENLFCSEEMPSSDDE        115
BK      KMEQDVKVAHQPD--FG-TWSSSEVPTYGTEEWESWWSSFNEKWDEDLFCHEDMFASDEE        117
JC      KMEQGVKVAHQPD--FG-TWNSSEVPTYGTDEWESWWNTFNEKWDEDLFCHEEMFASDDE        117
KI      KLQDSVSSVHDLNEEEDNIWQSSQIPTYGTPDLDEWWSQFNTYWEEELRCNESMPSSPKR        120
WU      KLQECVSTVHQLNEEEDEVWSSSQIPTYGTPDWDYWWSQFNSYWEEELRCNEEMPKSPGE        120
        *::: *. .*: :   .  *.::::***** : : **. **    *:* * *.*  *  .

SV      AT----------ADSQHSTPPKKKR--KVEDPKDFPSELLSFLSHAVFSNRTLACFAIYT        163
BK      AT----------ADSQHSTPPKKKR--KVEDPKDFPSDLHQFLSQAVFSNRTLACFAVYT        165
JC      NT-----------GSQHSTPPKKKK--KVEDPKDFPVDLHAFLSQAVFSNRTVASFAVYT        164
KI      S------APEEEPSCSQATPPKKK--HAFDASLEFPKELLEFVSHAVFSNKCITCFVVHT        172
WU      TPTKRTREDDEEPQCSQATPPKKKKDNATDASLSFPKELEEFVSQAVFSNRTLTAFVIHT        180
                          ..::******     :   .** :*  *:*:*****: ::.*.::*

SV      TKEKAALLYKKIMEKYSVTFISRHNSYNHNILFFLTPHRHRVSAINNYAQKLCTFSFLIC        223
BK      TKEKAQILYKKLMEKYSVTFISRHMCAGHNIIFFLTPHRHRVSAINNFCQKLCTFSFLIC        225
JC      TKEKAQILYKKLMEKYSVTFISRHGFGGHNILFFLTPHRHRVSAINNYCQKLCTFSFLIC        224
KI      TREKGEVLYKKLLQKYQCSFISKHAFYNTVLIFFLTPHKHRVSAINNFCKGHCTVSFLFC        232
WU      TKEKAETLYKKLLSKFKCNFASRHSYYNTALVFILTPFRHRVSAVNNFCKGYCTISFLFC        240
        *:**.  ****::.*:. .* *:*   .  ::*:***.:*****:**:.:  **.***:*

SV      KGVNKEYLMYSALTRDPFSVIEESLPGGLKEHDFNPEEAE---ETKQVSWKLVTEYAMET        280
BK      KGVNKEYLLYSALTRDPYHTIEESIQGGLKEHDFNPEEPE---ETKQVSWKLITEYAVET        282
JC      KGVNKEYLFYSALCRQPYAVVEESIQGGLKEHDFNPEEPE---ETKQVSWKLVTQYALET        281
KI      KGVNNPYGLYSRMCRKPFNLCEENIPGGLKENEFNPEDLFGEPKEPSLSWNQIANFALEF        292
WU      KGVNNAYGLYSRMTRDPFTLCEENIPGGLKENDFKAEDLYGEFKD-QLNWKALSEFALEL        299
        ****: * :** : *.*:   **.: *****::*: *:     :  .:.*: ::::*:*

SV      KCDDVLLLLGMYLEFQYSFEMCLKCIKKEQPSHYKYHEKHYANAAIFADSKNQKTICQQA        340
BK      KCEDVFLLLGMYLEFQYNVEECKKCQKKDQPYHFKYHEKHFANATIFAESKNQKSICQQA        342
JC      KCEDVFLLMGMYLDFQENPQQCKKCEKKDQPNHFNHHEKHYYNAQIFADSKNQKSICQQA        341
KI      DIDDVYYLLGSYIRFATKPEECEKCSKNDDATHKRVHVQNHENAVLLQESKSLKNACTQA        352
WU      GIDDVYLLLGLYLQLSIKVEECEKCNSNEDATHNRLHMEHQKNALLFSDSKSQKNVCQQA        359
          :**  *:* *: :   . : * ** .:::  *  . * ::  ** :: :**. *. * **

SV      VDTVLAKKRVDSLQLTREQMLTNRFNDLLDRMDIMFGSTGSADIEEWMAGVAWLHCLLPK        400
BK      VDTVLAKKRVDTLHMTREEMLTERFNHILDKMDLIFGAHGNAVLEQYMAGVAWLHCLLPK        402
JC      VDTVAAKQRVDSIHMTREEMLVERFNFLLDKMDLIFGAHGNAVLEQYMAGVAWIHCLLPQ        401
KI      IDRVIAERRYNCVTLTRKKLLTKRFKKLFNEMDKIVV--GERKILLYMASIAWYTGLNKK        410
WU      IDVVIAKRRVDSLNMSREDLLARRFEKILDKMDKTIK--GEQDVLLYMAGVAWYLGLNGK        417
        :* * *::* : : ::*:.:*..**: :::.**   .    *.  :  :**.:**   *  :

SV      MDSVVYDFLKCMVYNIPKKRYWLFKGPIDSGKTTLAAALLELCGGKALNVNLPLDRLNFE        460
BK      MDSVIFDFLHCIVFNVPKRRYWLFKGPIDSGKTTLAAGLLDLCGGKALNVNLPMERLTFE        462
JC      MDTVIYDFLKCIVLNIPKKRYWLFKGPIDSGKTTLAAALLDLCGGKSLNVNMPLERLNFE        461
KI      IDELVVRFLKLIVDNKPKHRYWLFKGPINSGKTTLATALLNLCGGKALNINIPSEKLPFE        470
WU      IDELVYRYLKVIVENVPKKRYWVFKGPINSGKTTVAAALLDLCGGKALNINIPADRLNFE        477
        :* ::  :*: :* * **:***:*****:*****:*:.**:*****:**:*:* ::* **

SV      LGVAIDQFLVVFEDVKGTGGESRDLPSGQGINNLDNLRDYLDGSVKVNLEKKHLNKRTQI        520
BK      LGVAIDQYMVVFEDVKGTGAESKDLPSGHGINNLDSLRDYLDGSVKVNLEKKHLNKRTQI        522
JC      LGVGIDQFMVVFEDVKGTGAESRDLPSGHGISNLDCLRDYLDGSVKVNLERKHQNKRTQV        521
KI      LGVALDQYMVVFEDVKGQIGIEKQLPSGNGVNNLDNLRDYLDGCVEVNLEKKHVNKRSQI        530
WU      LGVAIDQFTVVFEDVKGQVGDNKLLPSGNGMSNLDNLRDYLDGSVKVNLEKKHLNKRSQI        537
        ***.:**: ********   . .: ****:*:.*** *******.*:****:** ***:*:

SV      FPPGIVTMNEYSVPKTLQARFVKQIDFRPKDYLKHCLERSEFLLEKRIIQSGIALLLMLI        580
```

```
BK      FPPGLVTMNEYPVPKTLQARFVRQIDFRPKIYLRKSLQNSEFLLEKRILQSGMTLLLLLI        582
JC      FPPGIVTMNEYSVPRTLQARFVRQIDFRPKAYLRKSLSCSEYLLEKRILQSGMTLLLLLI        581
KI      FPPGIVTMNEYCIPETVAVRFEKTVMFTIKRNLRESLEKTPQLLSQRILHSGIAMLLLLI        590
WU      FPPGIVTMNEYLVPATLAPRFHKTVLFTPKRHLKESLDKTPELMVKRVLQSGMCILIMLI        597
        ****:****** :* *:  ** : : *  *  *:..*. :  *: :*:::**: :*::**

SV      WYRPVAEFAQSIQSRIVEWKERLDKEFSLSVYQKMKFNVAMGIGVLDWLRNSDDDDEDSQ        640
BK      WFRPVADFATDIQSRIVEWKERLDSEISMYTFSRMKYNICMGKCILDITREEDSETEDSG        642
JC      WFRPVADFAAAIHERIVQWKERLDLEISMYTFSTMKANVGMGRPILDFPREEDSEAEDSG        641
KI      WYRPVSDFDEEIQSNVVYWKEVLDNYIGLTEFATMQMNVTNGKNILEKWFE---------        641
WU      WCRPVSDFHPCIQAKVVYWKELLDKYIGLTEFADMQMNVTNGCNILEKHNA---------        648
        * ***::*   *: .:* *** **  :.:  :  *: *:  *  :*:

SV      ENADKNEDGGEKNMEDSGHETGIDSQSQGSF------QAPQSSQSVHDHNQPYHICRGFT        694
BK      ------------------HGSSTESQSQCSSQVSDTSAPAEDSQRSDPHSQELHLCKGFQ        684
JC      ------------------HGSSTESQSQCFSQVSEASGAD------TQENCTYHICKGFQ        677
KI      ------------------------------------------------------------        641
WU      ------------------------------------------------------------        648

SV      CFKKPPTPPPEPET    708
BK      CFKRPKTPPPK---    695
JC      CFKKPKTPPPK---    688
KI      --------------    641
WU      --------------    648
```

Deltapolyomavirus

```
H6          MDRLLAREEVKELMNLIGLSMACWGNLPLMQQKIRLACKKYHPDKGGDPEKMQRLNVLKE        60
H7          MDKLLGRDEVKELMELIGLNMACWGNLPLIQHKVRLASKKYHPDKGGDPQKMQRLNVLKD        60
Q           MDRLLSRDEVNELMQLIGLSMSNWGNLPLIQHKVREACKKHHPDKGGDPEKMQRLNVLKD        60
SV          MDKVLNREESLQLMDLLGLERSAWGNIPLMRKAYLKKCKEFHPDKGGDEEKMKKMNTLYK        60
H10         MDRVLSRDEVKELMALLSLNTAAWGNIPLMQYKYRQTCLKLHPDKGGDGEKMKRLNELFS        60
STL         MDQALSRAEAKELMGLLGLPEDSWGNVPLISYRYRQKSKIYHPDKGGNEETMKRLTELYS        60
            **: * * *  :** *:.*    ***:**:       .   ******: :.*:::. * .

H6          KLNATLRDQMSSS--PTWCFSS-EVRPPPQYGSPGWEQWWADFNRGWDDEDLYCDEHLSA        117
H7          KLQATLRDQRSGS--PMWHYSSDEVRPPPPYGSPAWDQWWQDFNKGWD-EDLYCTEELSS        117
Q           KFAATMRDQSSGN--PIWHFSSEEVRPPPPYGTPEWDKWWHDFNRGWD-EDLYCTEELSA        117
SV          KMEDGVKYAHQPDFGGFW-----DATEIPTYGTDEWEQWWNAFNE----ENLFCSEEMPS        111
H10         KMYTTIEKLRRE-GEVYF-----PAKGNPTYGTPEWDQWWEEFNRGWD-EDLSCNESFAP        113
STL         RMQNTLQNLRSS-SENEN-----VFPPGGQYGTPAWEQWWEEFNQPFE-DDLTCNESFNC        113
            ::   :.                       **:  *::**  **.    ::* * * :

H6          SEEEDN-----VD--------PGEGNSQDSKYSCTPPKKRKPNPAPNDFPSCLHDYLSHA        164
H7          SDEEEPAASASVN--------PEEGCSQDSKYSATPPKQKKPNPAPQDFPECLSEFLSHA        169
Q           SDEEQTA----ED--------PEEGCSQNSKYSATPPKQRKPNPAPQDFPECINEYLSHA        165
SV          SDDEATADSQH----------------------STPPKKKRKVEDPKDFPSELLSFLSHA        149
H10         SDEEEPGPSQSASQTANDTNTPKKRPRESSSNSTCTPPKRPRNFNPVDFPEVLLEFLSNA        173
STL         SDDEGTSASQK-------------RKFPDSSTQNSTPPKKNKPADPTDFPAELETFLSHA        160
            *::*                                * ::     * ***   :  :**:*

H6          TLGNKCYTCFVSYTTLEKWETLYDKLQSAFNAVFTGAYKCNDNTGAILYCITPRRHRVSA        224
H7          TLGNKCYTCFLCYTTYEKSMLLYEKLGVEFNALFIGAYNCVDGSGALVFFISGSRHRVSA        229
Q           TLGNKCYNCFVCYTTMEKSLMLYDKLNNEFNALFIGNYKCNDGSGSIVYMITGSRHRPSA        225
SV          VFSNRTLACFAIYTTKEKAALLYKKIMEKYSVTFISRHNS--YNHNILFFLTPHRHRVSA        207
H10         IFSNKTLNSFVLYTTREKGQFLYEKVPLKFKAMFYSLHEF--DGDSLLFLLLSGKHRVSA        231
STL         VFSNKTSNCFCIYTTIEKGNELYTVIGPKFKSMFISCHSY--NTCCILFMILAGKHRVSA        218
             :.*:   .*  *** **   **  :   :.  * . :.       ::: :   :** **

H6          MLNALSKCCTISFLLIKAVLKSAECYMALQGDEFTVIQESKAEGLHSYDFQEGSK--KEE        282
H7          ILNACKKHCTVSFIMVKAVLKNAECYKALQDSKFAVLRESKEGGLHSYDFQEASK--KDD        287
Q           ILNASKKYCTVSFSLVKAVLKNAECYKALQGPNFTVIRESREGGLHSYDFQEASK--KDD        283
SV          INNYAQKLCTFSFLICKGVNKEYLMYSALTRDPFSVIEESLPGGLKEHDFNPEEAEETKQ        267
H10         IKNYCSNLCTVSFLLVKGCLKAYECYYALCKTPFKLIKQSQEHGLSKTDFCEEEK--DKV        289
STL         LKNFCAALCSISFVLVKSCLKPYECYYRLCSAPFAVTKQSRPEGLSQAEFMEQEN-SKPS        277
            : *     *:.** : *.  *    *  *    * : .:*   ** . :*   .

H6          CDWNQVASFASDTDLTDCLALLGYYIEFANDPASCMKCKKGVKV--HKHHEVHFHNAQLF        340
H7          CDWNFVADFAADMELTDVLLIMGYYMEFATEPSLCPKCLKSVKA--HQHHEKHWANAKLF        345
Q           CDWNAVAEFALANDLTDPLLIMGYYLEFAAEPSLCQKCKKGVKA--HKCHELQWSNAKLF        341
SV          VSWKLVTEYAMETKCDDVLLLLGMYLEFQYSFEMCLKCIKKEQPSHYKYHEKHYANAAIF        327
H10         VNWQQICEFAVEVQCEDPLLLMGMLLDFAKDVEGCSKCEQKKLKHHYKFHEAQNINSKLF        349
STL         VNWQQICEFAVQFNCEDPLLLMGIYLDFSESPENCEKCKT-ELKHHSQFHEKEYNNAKLF        336
             .*: : .:*   .  * * ::*  ::*  .   * **         : ** .  *: :*

H6          KAAKNQKSIAQQACDRVAAQRRVLMLESTRQDLVLQAFKKQFKILAEQY--AGGVEITQL        398
H7          KTAKNQKGIAQQAADRVLAARRVLMMESTRKDLMVMSFKKQFKVLAEQF--GGGVEITQL        403
Q           KSAKNQRGIATQAADRVLSARRVMMIESTRVDLMVMAFKKQFQVLNDQF--AGGVEITQL        399
SV          ADSKNQKTICQQAVDTVLAKKRVDSLQLTREQMLTNRFNDLLDRMDIMFGSTGSADIEEW        387
H10         KDCKNQKTICQQATDWVTAQRRLLILESTREHLLVLRFKHMFEKMEDI---CGEVEICQY        406
STL         RDSKTQKTLCQQACDWVCAKRRVLILESTREDLLVIRFKQVLKEMQDI---AGEVEILRY        393
              .*.*: :. ** * * : :*:  :: ** .::   *:. :. :       * .:* .

H6          LGAVAWLDCLQPSFTTKLKEILSILTENIPKKRNVLFKGPINSGKTTLAAAILDLVGGVS        458
H7          IGAVAWLDCLLPQFTIKIKEMLSYLVENTPKRRNLLFKGPINSGKTTLAAAILDLLGGVA        463
Q           LGAVAWLDCLMPSFTTKLKEMLTLLVQNYAKKRNLLFKGPINSGKTTVAAGIMDLLGGVA        459
SV          MAGVAWLHCLLPKMDSVVYDFLKCMVYNIPKKRYWLFKGPIDSGKTTLAAALLELCGGKA        447
H10         MAGVAWLSLLMPHFDEIILFIIKAMTENVPKRRYVLFKGPINSGKTTVAAAILDLLGGKT        466
STL         MAGVAWLSLLFNHFDDIVLEIIRTMVINTPKRRYFLFKGPINSGKTTVAAAILDLLGGRT        453
            :..****  *   :   :  ::  :. *  *:*  ******:*****:**.:::* ** :

H6          LNVNCTPDKINFELGCAIDKFMCVIEDVKGTP-MANTNLTQGCGMTNLDNLRDYLDGCVP        517
H7          LNVNCSSDKINFELGCAIDKYMVVIEDVKGTP-LPNTDLPSGVGMANLDNMRDYLDGCVP        522
```

```
Q        LNVNCSSDKINFELGCAIDKMLVVFEDVKGQP-LPNTDLPAGVGMANLDNLRDHLDGCVP      518
SV       LNVNLPLDRLNFELGVAIDQFLVVFEDVKGTG-GESRDLPSGQGINNLDNLRDYLDGSVK      506
H10      LNVNCPPDKLAFEIGCAIDEYMVVFEDVKGQNEGSNSSLTPGMGMSNLDNLRDHLDGCVK      526
STL      LNINCPPEKVNFELGCAIDEFMVVFEDVKGQTEGK-TNLTSGMGMNNLDSLRDHLDGCVK      512
         **:*   ::: **:* ***: : *:*****       .*  * *: ***.:**:***.*

H6       VNMERKHLNKVSQLFPPSVITCNEYVIPCTVKARIARGYYFLHKPCLQKCLKD-CVLMSK      576
H7       VNLERKHINKTSQLFPPCIITCNEYAIPTTVKARVAKGYYFLHKPGLKKSLDANPILMKK      582
Q        VNLERKHTNKVSQLFPPCIITCNDYAIPRTVKARVAKGYYFIHKPNLKKCLDVNPILMQK      578
SV       VNLEKKHLNKRTQIFPPGIVTMNEYSVPKTLQARFVKQIDFRPKDYLKHCLERSEFLLEK      566
H10      VNLEKKHVNKKSQIFPPGIITMNDYFIPPTLQARMIKTINFRPKLFLRNSLEKNSELLRK      586
STL      VNLEKKHLNKRSQIFPPGIITMNEYNVPLTILARMVKVINFRPKHYLKKSLEVNNELLHR      572
         **:*:** ** :*:*** ::* *:* :* *: **. :   *  *  *::.*.    *: :

H6       RLLQKGTTLLAALIWWEPVEDFMEELQEDVVNWKQTFERWVSFGMYQTMKENILAGIDPF      636
H7       RLLQKGCTLLAALIWWEPVSDFVEEIQEEVVNWKQTFEQWVSYGMFQTMKENILSGKDPF      642
Q        RLLQKGVTLLAALIWWEPVSEFVEEIQEDVVNWKQTFERWVTYGMYQDMKQNILAGKDPF      638
SV       RIIQSGIALLLMLIWYRPVAEFAQSIQSRIVEWKERLDKEFSLSVYQKMKFNVAMGIGVL      626
H10      RIVQSGVTLLLLLCWWQPVIAFHPEIHDNVRYWKETIEKYVPFGMYHDIRRNIESGEDPL      646
STL      RIVQSGKTLLMLLMWWQPVKVFHSSIHEDVKIWKETLTKYVSIGMFHDMQRNIQNGDDPL      632
         *::*.* :**  * *:.**  *  .::. : **: : : .  .::: :: *:  * . :

H6       TNVLVDESFVQPQEND-ETN---------DSTQESGIGSMHSM-----------------      669
H7       EGVLINDPTE---ENT-RET---------QESTESGIGSMNN------------------      671
Q        YGVIMSDIN----EIV-EET---------QESTESGVGSMET------------------      666
SV       DWLRNSDDDDEDSQENADKNEDGGEKNMEDSGHETGIDSQSQGSFQAPQSSQSVHDHNQP      686
H10      KDILICVDADEDTQQDSGINSQ--------------------------------------      668
STL      KNILICEDTENNETQDSAFCTQDSDNE---------------------------------      659
           :

H6       ----------------------   669
H7       ----------------------   671
Q        ----------------------   666
SV       YHICRGFTCFKKPPTPPPEPET   708
H10      ----------------------   668
STL      ----------------------   659
```

Reference

[1] Madeira F, Pearce M, Tivey ARN, Basutkar P, Lee J, Edbali O, Madhusoodanan N, Kolesnikov A, Lopez R. Search and sequence analysis tools services from EMBL-EBI in 2022. Nucleic Acid Res. 2022; 50: W276-279. DOI: 10.1093/nar/gkac240

Supplementary Figure S2. Alignment of the HPyV and SV40 sTAg. The Alphapolyomaviruses are shown in pink, the Betapolyomaviruses in yellow, and the Deltapolyomaviruses in turquoise. Conserved and partially conserved S, T and Y are highlighted in red, green, and blue, respectively.

```
SV40      MDKVLNREESLQLMDLLGLERSAWGNIPLMRKAYLKKCKEFHPDKGGDEEKMKKMNTLYK     60
BKPyV     MDKVLNREESMELMDLLGLERAAWGNLPLMRKAYLRKCKEFHPDKGGDEDKMKRMNTLYK     60
JC        MDKVLNREESMELMDLLGLDRSAWGNIPVMRKAYLKKCKELHPDKGGDEDKMKRMNFLYK     60
KI        MDKTLSREEAKQLMQLLCLDMSCWGNLPLMRRQYLVKCKEYHPDKGGNEESMKLLNSLYL     60
WU        MDKTLSRNEAKELMQLLGLDMTCWGNLPLMRTKYLSKCKEFHPDKGGNEEKMKKLNSLYL     60
NJ        MEKVLEKSDKEMLIELLGIPRYAYGNFPIMKTAYKRASKIYHPDKGGSSEKMMLLNSLWQ     60
TS        MDKFLSREESLELMDLLQIPRHCYGNFALMKINHKKMSLKYHPDKGGDPEKMSRLNQLWQ     60
MC        MDLVLNRKEREALCKLLEIAPNCYGNIPLMKAAFKRSCLKHHPDKGGNPVIMMELNTLWS     60
H6        MDRLLAREEVKELMNLIGLSMACWGNLPLMQQKIRLACKKYHPDKGGDPEKMQRLNVLKE     60
H7        MDKLLGRDEVKELMELIGLNMACWGNLPLIQHKVRLASKKYHPDKGGDPQKMQRLNVLKD     60
Q         MDRLLSRDEVNELMQLIGLSMSNWGNLPLIQHKVREACKKHHPDKGGDPEKMQRLNVLKD     60
H9        MDQTLSLEERNELMDLLQLTRAAWGNLSLMKKAYKTVSKIYHPDKGGNPEKMQRLNELFQ     60
H10       MDRVLSRDEVKELMALLSLNTAAWGNIPLMQYKYRQTCLKLHPDKGGDGEKMKRLNELFS     60
STL       MDQALSRAEAKELMGLLGLPEDSWGNVPLISYRYRQKSKIYHPDKGGNEETMKRLTELYS     60
          *:  *   :   *  *: :    :**. ::       .   ******.   *  :. *

SV40      KMEDGVKYAHQPD--FGGFWDATEVFASS----LNPGV-------------DAMYCKQWP     101
BKPyV     KMEQDVKVAHQPD--FGT-WSSSEV-CAD----FPLCP-------------DTLYCKEWP     99
JC        KMEQGVKVAHQPD--FGT-WNSSEV-GCD----FPPNS-------------DTLYCKEWP     99
KI        KLQDSVSSVHDLNEEEDNIWQSSQVYCKDLCCN----KFRLVNAIYGE-YYEAYIMKQWD     115
WU        KLQECVSTVHQLNEEEDEVWSSSQVECTELCCNFPPRKYRLVGEVYGD-VFEEYILKDWD     119
NJ        KFQEGLIEVRDSE---VCQVSFSDCY-------------DSSLLKCCSPKVFHELF-LRSP     104
TS        KLQEGIYNARQEF---PTSF-SSQVGSW-----YWEANLISLKEYFGKKKYDENVIKHWP     111
MC        KFQQNIHKLRSDF---SMFDEVS--TKF-----PW-EEYGTLKDYMQS-GYNARFCRGPG     108
H6        KLNATLRDQMSSS---PTWCFSSEVSDD------W-GIPLTVGEFLGP-EFHKKKVWDFC     109
H7        KLQATLRDQRSGS---PMWHYSSDEVSF------W-DIELTVGEFLGP-EFNRKKVWNYN     109
Q         KFAATMRDQSSGN---PIWHFSSEEVSF------W-DLQLTVGEFLGV-EFNRKKLWNFE     109
H9        KLQVTLLEIRSNC---GSSS--SQVAWY-----FWDENFRTLGAFLGE-KFNQRIIGGYP     109
H10       KMYTTIEKLRRE-----GEVYFPAKVGY------FIDDVVTLGDVLGP-SFEEKIIYIWP     108
STL       RMQNTLQNLRSSS---ENENVFPPVRML------LLSDTFTLGDLLGP-QFESKVIFIWP     110
          ::   :                                             .

SV40      ECAKKMS-ANCICLLCLLRMKHENRK----LYRKDPLVWVDCYCFDCFRMWFGLDLC-EG     155
BKPyV     ICSKKPS-VHCPCMLCQLRLRHLNRK----FLRKEPLVWIDCYCIDCFTQWFGLDLT-EE     153
JC        NCATNPS-VHCPCLMCMLKLRHRNRK----FLRSSPLVWIDCYCFDCFRQWFGCDLT-QE     153
KI        VCIHGYN-HECQCIHCILSKYHKEKY----KIYRKPPVWIECYCYKCYRECFFFPIS-MQ     169
WU        ICLKGFY-YLCNCFYCFLDKRHKQKY----KIFRKPPMWIECYCYRCYREWFGFEIS-AE     173
NJ        QCLLKGP-TSCSCITSCLYNQHRQIK---LCGKKRCLTWGNCFCFSCFILWFGLRET-WK     159
TS        QCAEKAL-KECKCLTCKIGLQHYVYK---QMHQKKCVVWGECFCYKCYCAWFGEDLYCLD     167
MC        CMLKQLRDSKCACISCKLSRQHCSLK---TLKQKNCLTWGECFCYQCFILWFGFPPT-WE     164
H6        LCVQQGI-SSCKCLHCLLKKEHKKQV---EINLGKPTIWGKCWCYKCYCLWFGLPVE-AD     164
H7        LCVVQGL-RACCCIHCILKRKHKKKAKEYAKDHRGPLLWGKCWCFDCYLDWFGVERS-EE     167
Q         LCVLQGL-RACCCLHCLLRRKHKKLAKQMAKDQKGPLVWGHCWCFQCYLQWFGEDKN-KE     167
H9        DCITYN-KPSCCCIVCLLKQQHKSTK---INKKKPCLVWGECFCYKCYLLWFGFPED-FT     164
H10       LCASDLLRHKCGCVCCLLKKQHRNDK---LAKQKQCLVWGECFCYKCFLLWFGQEFG-YT     164
STL       TCAKCRYRTFCKCICCILKRQHDEIK---QVRKKPCVTWGECYCFDCFLLWFGCDFT-KT     166
                    * *. . :   *                * .*:*  *:   *

SV40      TLLLWCDIIGQTTYRDLKL-----------------------     174
BKPyV     TLQWWVQIIGETPFRDLKL-----------------------     172
JC        ALHCWEKVLGDTPYRDLKL-----------------------     172
KI        TFFFWKVIIFNTEIRAVQPLLR--------------------     191
WU        TFFYWKKIIFLTTMQGVGLTR---------------------     194
NJ        TFEIWKHVIAQMPAALLQLSPSLF------------------     183
TS        SLWAWSCIVGEVDFHLVNLYLRVNQGFNWGK-----------     198
MC        SFDWWQKTLEETDYCLLHLHLF--------------------     186
H6        SFMWWTHIIYQSPLDWLGITEKLIWW----------------     190
H7        SFMWWSHIIFQTPMDVLNLWGQLNLL----------------     193
Q         SFEWWTQIIYGTQMDVINIWGQINLL----------------     193
H9        SFNYWTLLMRNMDLSLLRLWTELGF-----------------     189
H10       SFFWWKHIMHNTEFDLLCLLGELILWVSYFSFILGKSHLWDS     206
STL       SLHAWKHVMFNLDLDLLMFKQLNLVSFSF-------------     195
          ::  *   :       :
```
